# A multi-targeted computational drug discovery approach for repurposing tetracyclines against monkeypox virus

**DOI:** 10.1038/s41598-023-41820-z

**Published:** 2023-09-04

**Authors:** Thamir A. Alandijany, Mai M. El-Daly, Ahmed M. Tolah, Leena H. Bajrai, Aiah M. Khateb, Geethu S. Kumar, Amit Dubey, Vivek Dhar Dwivedi, Esam I. Azhar

**Affiliations:** 1 Special Infectious Agents Unit-BSL3, King Fahd Medical Research Center, King Abdulaziz University, 21362 Jeddah, Saudi Arabia; 2 Department of Medical Laboratory Sciences, Faculty of Applied Medical Sciences, King Abdulaziz University, 21362 Jeddah, Saudi Arabia; 3 Department of Medical Laboratory Technology, Faculty of Applied Medical Sciences, King Abdulaziz University, Rabigh, Saudi Arabia; 4 Biochemistry Department, Faculty of Sciences, King Abdulaziz University, Jeddah, Saudi Arabia; 5 Department of Medical Laboratory Technology, College of Applied Medical Sciences, Taibah University, 42353 Madinah, Saudi Arabia; 6 Department of Life Science, School of Basic Science and Research, Sharda University, Greater Noida, Uttar Pradesh India; 7 Computational Chemistry and Drug Discovery Division, Quanta Calculus, Greater Noida, India; 8 Bioinformatics Research Division, Quanta Calculus, Greater Noida, India; 9 Center for Global Health Research, Saveetha Medical College and Hospitals, Saveetha Institute of Medical and Technical Sciences, Saveetha University, Chennai, India

**Keywords:** Virtual drug screening, Virtual screening

## Abstract

Monkeypox viral infection is an emerging threat and a major concern for the human population. The lack of drug molecules to treat this disease may worsen the problem. Identifying potential drug targets can significantly improve the process of developing potent drug molecules for treating monkeypox. The proteins responsible for viral replication are attractive drug targets. Identifying potential inhibitors from known drug molecules that target these proteins can be key to finding a cure for monkeypox. In this work, two viral proteins, DNA-dependent RNA polymerase (DdRp) and viral core cysteine proteinase, were considered as potential drug targets. Sixteen antibiotic drugs from the tetracycline class were screened against both viral proteins through high-throughput virtual screening. These tetracycline class of antibiotic drugs have the ability to inhibit bacterial protein synthesis, which makes these antibiotics drugs a prominent candidate for drug repurposing. Based on the screening result obtained against DdRp, top two compounds, namely Tigecycline and Eravacycline with docking scores of − 8.88 and − 7.87 kcal/mol, respectively, were selected for further analysis. Omadacycline and minocycline, with docking scores of − 10.60 and − 7.51 kcal/mol, are the top two compounds obtained after screening proteinase with the drug library. These compounds, along with reference compounds GTP for DdRp and tecovirimat for proteinase, were used to form protein–ligand complexes, followed by their evaluation through a 300 ns molecular dynamic simulation. The MM/GBSA binding free energy calculation and principal components analysis of these selected complexes were also conducted for understanding the dynamic stability and binding affinity of these compounds with respective target proteins. Overall, this study demonstrates the repurposing of tetracycline-derived drugs as a therapeutic solution for monkeypox viral infection.

## Introduction

Monkeypox viral infection has become a worldwide issue since many incidences in several non-endemic nations were recorded in May 2022. The outbreak was first reported in 11 countries in Central and West Africa, then exported to other parts of the world, affecting 110 countries^1,2^. This zoonotic viral infection caused by the double-stranded DNA monkeypox virus (MPXV) belongs to the Orthopoxvirus genus of the Poxviridae family, which includes Variola, Camelpox, Cowpox, Akhemeta, and Alaskapox viruses^3^. Monkeypox viral infection shows considerable similarity to smallpox due to smallpox-like exanthema. However, monkeypox mortality rate and clinical symptoms are lower than smallpox^4^. Mammals like squirrels, dormice, rodents, rabbits and various primates are some natural reservoirs of MPXV. Viral transmission is caused by direct contact with an infected animal’s bite, scratches, or consumption of bush meat. In humans, viral transmission occurs through contact with body fluids, respiratory droplets, and lesions of the infected person or animals^4,5^.

**Figure 1:**
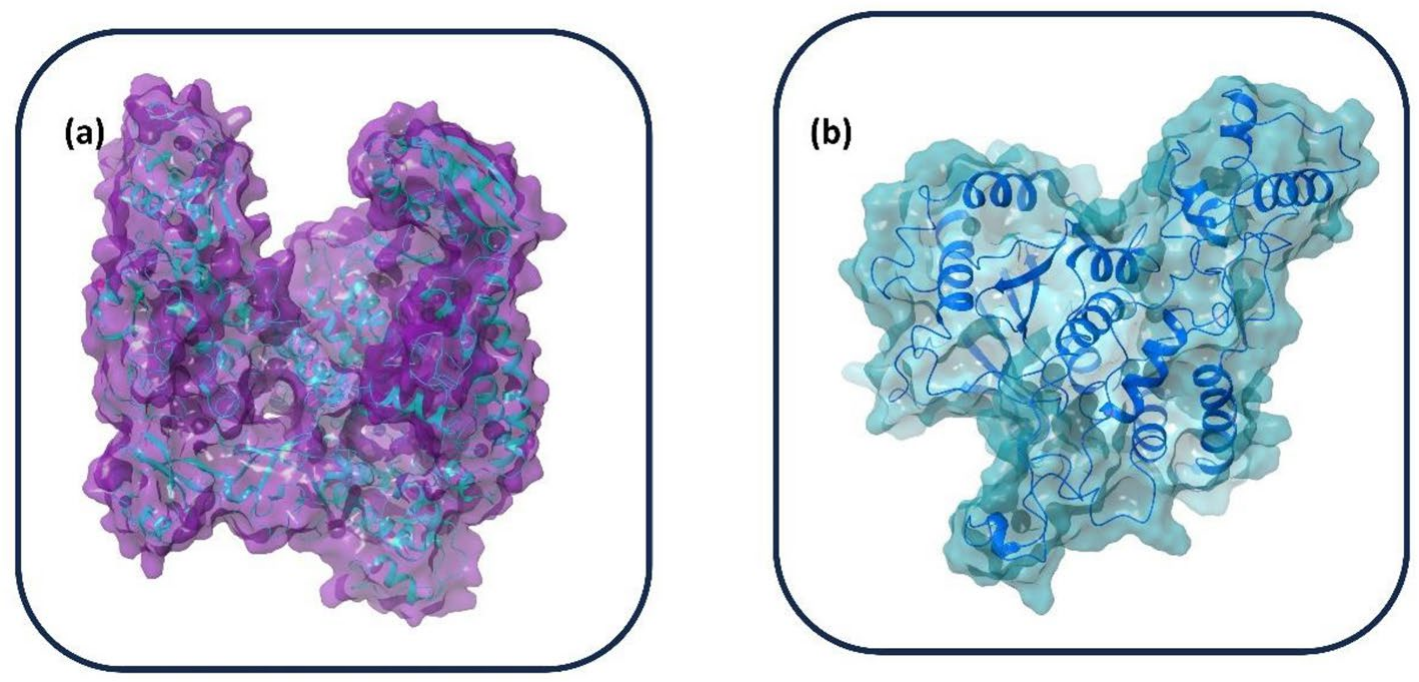
3D structure of monkepox virus (**a**) DNA-dependent RNA polymerase (DdRp) and (**b**) cysteine proteinase modelled protein.

MPXV has a brick-shaped structure, with a genome size of 196,858 bp, and encodes approximately 200 proteins^6^. The genes are closely packed, and those present in the terminal end encode proteins responsible for pathogenesis. Housekeeping genes are present in the conserved central regions and contribute to the transcription and replication of the virus^7,8^. Even though the genome is arranged uniformly, the current viral outbreak is due to high mutation, especially the increase in DNA viruses; therefore, MPXV is under microevolution during human-to-human transmission^9,10^. The proteolytic development of the MPXV core proteins is necessary for generating infectious virions. Unlike other DNA viruses, MPXV replication cycle occurs in the cytoplasm of infected cells using a range of virus-encoded proteins, such as DNA-dependent RNA polymerase (DdRp) and cysteine proteinase^11^. DdRp is required by the infected host cell to transcribe the genome in its cytosol, and cysteine proteinase, also known as core protease, cleaves the precursor polyprotein^12,13^. These factors display the poxvirus cysteine protease and DdRp as potential pharmacological targets for producing novel therapeutic antivirals (Fig. 1).

MPXV-infected patients usually experience mild symptoms and may not require medication. But, if the patients are at risk of severe dehydration, they must be given proper medicine and supportive care^14^. JYNNEOS™ and ACAM_2000_® are licensed vaccines approved for MPXV^15^. Antiviral drugs, such as Brincidor, Tecovirimat and Clodivir, showed promising results against monkeypox viral infection when tested in animal models. Tecovirimat, which the FDA approves for smallpox treatment, is considered a potential drug for clinical use against monkeypox treatment^16^. So, only a limited number of FDA-approved antiviral drugs are utilised for treating this viral disease, and there is a need for identifying potential antiviral drugs. Fludarabine, Norov-29 and bemnifosbuvir showed promising inhibitory effects against the MPXV DdRp protein during the in silico analysis^17,18^. TTP-6171 has shown inhibitory activity against the cysteine proteinase^19^. A series of microbial-derived natural products were also screened to identify potential cysteine proteinase inhibitors. Gallicynoic Acid F and H2-Erythro-Neopterin showed significant inhibitory action against the cysteine proteinase of MPXV^20^.

Finding novel, efficient, and secure remedies remain vital to combat the monkeypox outbreak that still lack specific medicines. The traditional way identifying new drug molecules is a tedious task also it a time-consuming process. Due to high spreading rate of this infection identification of effective drug molecule in a short time span is necessary^21^. The recent studies have proven that repurposing of FDA drugs using computational approaches has been an effective approach for drug identification for treatment of several diseases^22,23^. This investigation uses a thorough computational process to identify potential drug molecules against MPXV DdRp and proteinase.

## Methodology

### Computational modelling of protein structures

The three-dimensional (3D) structure protein structures of monkeypox are not predicted through experimental validation. Hence, the targets proteins, i.e., Monkeypox virus DNA-dependent RNA polymerase subunit rpo147 (Accession No: UTG36891.1) and viral core cysteine proteinase (Accession No: NP_536495.1) collected from GenPept database of GenBank gene products for computational modelling^24^. The MPXV DdRp primary sequence in FASTA format was used homology modelling of the protein using the Alphafold Colab v2.1.0^25^. The structure of cysteine proteinase was also modelled using the same approach. The structure was validated using the information available in the previously published paper by Dubey et al., where the cysteine proteinase was already modelled using the same protocol^20^. The Alphafold modelling tool uses a deep learning algorithm of the machine learning approach with the help of multiple sequences for predicting 3D protein structure with maximum accuracy from its amino acid sequence^26^.

### Protein structure validation using molecular dynamic (MD) simulation

The modelled structures of both target proteins were validated by performing molecular dynamic simulation for 300 ns using the free academic Desmond tool^27,28^. Both the modelled proteins were prepared with the help of the protein preparation wizard module of the Schrodinger suite. Using the system-building tool, an orthorhombic box was built to simulate the modelled proteins. The salt and the ion around the ligand binding site of the modelled proteins at 20 Å were removed. To the whole system, transferable intermolecular potential with 4 points (TIP4P) and sodium ions was added as counter ions, followed by immersion of the entire system into a virtually created water bath. The constant pressure was maintained throughout the MD simulation by maintaining a 0.002 ps time step for the anisotropic diagonal scaling. Also, the system’s temperature was gradually increased to 310 K, followed by a 20 psi NPT at 1 atm pressure, and the entire system was compressed to 1 g/cm^3^. The optimised potential for liquid simulation (OPLS-2005) force field present in the academic version of Desmond software was applied to the system for the MD simulation calculation^29^. To obtain suitable confirmations of the system, the Desmond Trajectory Clustering tool present in the Maestro platform was used to cluster the frames from each trajectory based on the obtained root mean square deviation (RMSD). The most recurring structures from the cluster were selected and used for binding site prediction and virtual screening.

### Binding site prediction and Grid Box generation

The selected monkeypox viral proteins' binding site was predicted using the Computed Atlas of Surface Topography of proteins (CASTp) server^30^. This server provides an online platform for predicting the geometric and topological characters of the protein structure, along with visualising the active pocket of the protein structure^30^. The binding site identified using this server was utilised for grid box generation. The dimension of the generated grid box in the x,y and z axis is 60 Å × 60 Å × 60 Å, and in the center, the coordinates are at 23.95 Å, − 7.71 Å, and − 11.58 Å. These coordinates were utilised for docking the MPXV proteins with tetracycline antibiotic compounds.

### Compound library and ligand preparation

Sixteen drug molecules belonging to the class of tetracycline antibiotics were retrieved from the PubChem database^31^. These drugs were combined to generate the compound library that will be utilised for virtual screening. These drugs or ligand molecules were prepared using the LigPrep tool of Schrodinger suit^32,33^. Ligand preparation using the Ligprep tool reduces the Lewis structure formation of the ligand molecules and removes defective traits of ligands to reduce the chances of computational error. It also helps to optimise the ligand molecules by stabilising and expanding the tautomeric structures, ionisation state and ligand structure confirmations^20^. For further analysis, these prepared compounds were taken for high-throughput virtual screening (HTVS) against the predicted MPXV protein structures.

### High-throughput virtual screening (HTVS) analysis

A hierarchical level of structure-based virtual screening was performed using the Glide module of the Schrödinger suit^34,35^. Herein, the compounds are screened out at three different levels based on precision and accuracy, i.e., high-throughput virtual screening (HTVS), standard precision (SP) and extra precision (XP). This hierarchical screening helps filter out the potential ligand poses using an advanced scoring function that preciously binds to the protein's active site^36^. Based on the result obtained from the three-level screening process of MPXV DdRp and cysteine proteinase proteins against the tetracycline antibiotics, the top compounds with the highest docking score for each protein were selected and underwent molecular docking.

### MD simulation analysis of the protein–ligand complex

The top protein–ligand complexes are further analysed through 100 ns MD simulation performed using the Desmond module^28^. The parameters for the MD simulation of the modelled protein were kept the same as for the MD simulation analysis of the protein–ligand complex. A detailed explanation of the procedure followed is mentioned in section "Protein structure validation using molecular dynamic (MD) simulation" of the methodology. The interaction diagram tool available in the free-academic Desmond software was used to study the intramolecular interaction in the protein–ligand complexes. The simulation trajectories for 300 ns of both proteins were evaluated, and the RMSD, RMSF and protein–ligand contact mapping were extracted from these trajectories for each complex.

### Binding free energy calculation

The free binding energy of the protein–ligand complex is calculated using Molecular mechanics with a generalised Born and surface area solvation (MM/GBSA) method. The Prime module of the Schrodinger suite was used for this calculation, combined with the OPLS-2005 force field under default parameters^29,37^. MM/GBSA method calculates the various components, such as ΔG_Bind,_ ligand strain energy e.t.c., for protein, ligand and protein–ligand complex.

The MM/GBSA binding free energy is derived from the equation.

$$\Delta {\text{G}}_{{{\text{MMGBSAbind}}}} = {\text{E}}^{{{\text{complex}}}} - \left( {{\text{E}}^{{{\text{complex}}/{\text{receptor}}}} + {\text{E}}^{{{\text{complex}}/{\text{ligand}}}} } \right)$$

### Principal component analysis (PCA)

Principal component analysis or PCA of each complex extracted from the MD simulation trajectories was performed using the bio3d R package^38^. This statistical technique reduces and identifies all the significant fluctuations experienced by the protein residues through covariance matrix analysis of the alpha-carbon (Cα) atoms in the protein. The eigenvectors with highest egienvalues are considers as principal components. Therefore, three major principal components are considered for this analysis. This method gives an insight about the dynamics of the protein by calculating these principal components.

### Ethical approval

This article does not contain any studies involving human participants or animals performed by any of the authors.

## Result and discussion

### HTVS analysis

The most recurring cluster obtained after the 100 ns MD simulation of the modelled DdRp and cysteine proteinase proteins of MPXV was used to obtain the middle structure of these proteins. Both of these proteins were virtually screened against tetracycline antibiotics to identify potential inhibitory drug molecules based on their binding affinity. A complete set of 16 tetracycline antibiotics were retrieved from the PubChem database for screening against the two selected MPXV proteins. Stereoisomers of these drugs were generated using the LigPrep tool, which was then filtered out with the help of the QikProp filter. Based on the docking score obtained after the three-tire screening, i.e., HTVS, SP and XP, the top two compounds for each protein were selected for further analysis. Two reference ligands were also selected and docked against the selected MPXV protein. GTP was considered as one of the reference ligand, which was docked with the DdRp MPXV protein, and in the case of MPXV proteinase, tecovirimat antiviral drug were used as reference compound for docking with cysteine proteinase.

**Figure 2:**
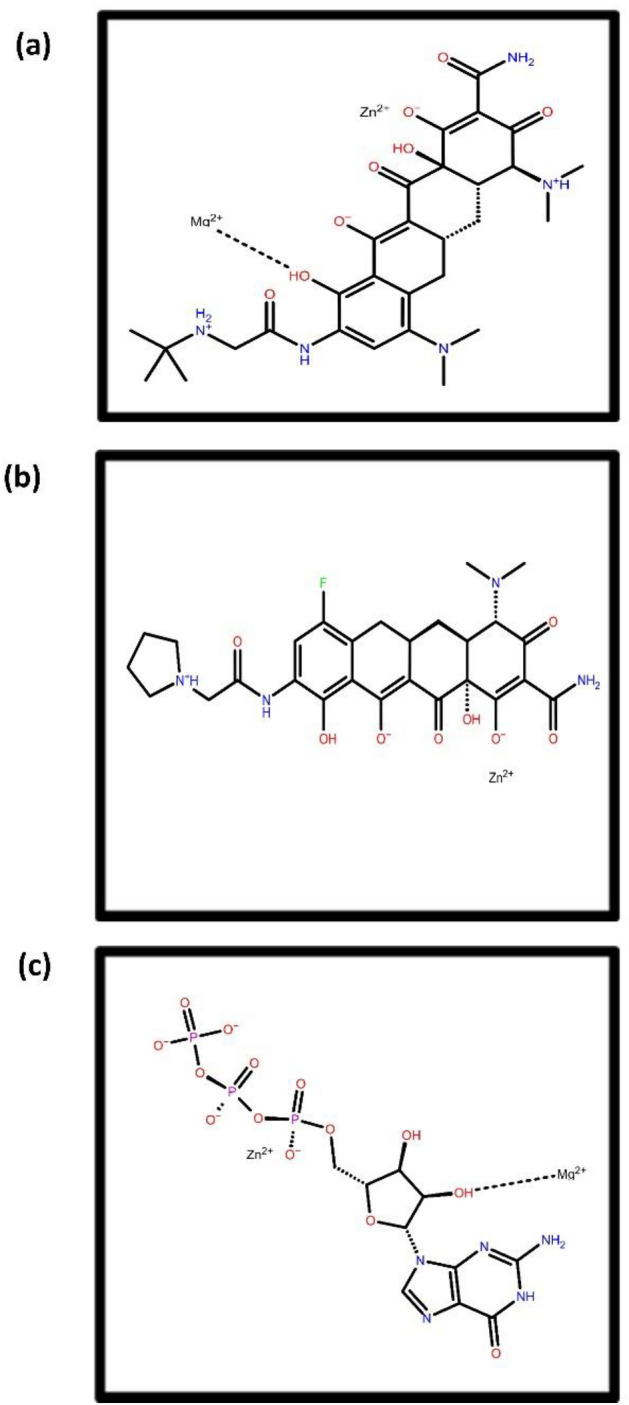
2D structure representation of selected top two drugs viz., (**a**) Tigecycline, (**b**) Eravacycline antibiotic drugs after screening against MPXV DdRp along with reference compound (**c**) GTP.

The top two compounds that showed the highest docking score when screened against MPXV DNA-dependent RNA polymerase are Tigecycline (PubChem CID54686904) and Eravacycline (PubChem CID54726192), having docking scores of -8.88 and -7.87 kcal/mol, respectively (Fig. 2, Supplementary Table S1). Tigecycline is a broad-spectrum glycylcycline antibiotic drug obtained from tetracycline for antimicrobial therapy. It is mainly used for the treatment of intravenous skin diseases. This drug prevents adding amino acids into the peptide chain and inhibits protein synthesis and bacterial growth^39^. Eravacycline is significantly used to treat gram-negative, gram-positive and facultative bacterial diseases. This also suppresses bacterial growth by inhibiting protein synthesis^40^.

**Figure 3:**
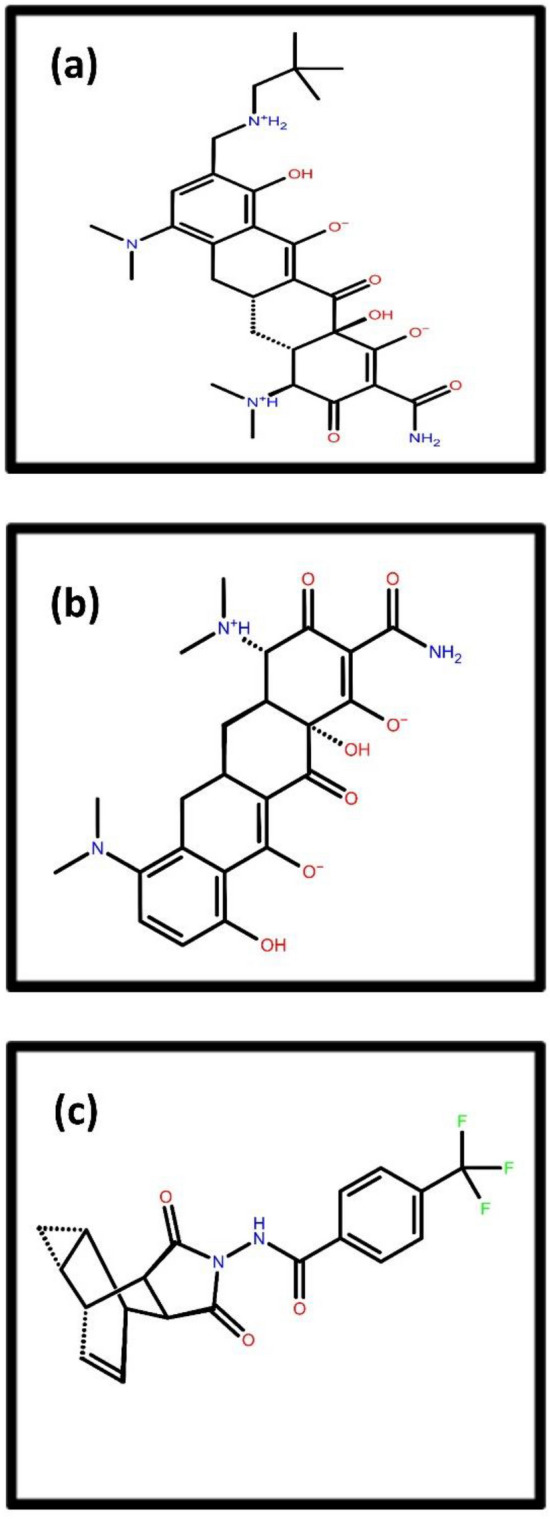
2D structure representation of selected top two drugs viz., (**a**) Omadacycline (**b**) Minocycline antibiotic drugs after screening against MPXV proteinase along with reference compound (**c**) Tecovirimat.

Omadacycline (PubChem CID 54697325) and Minocycline (PubChem CID 54675783) are two antibiotics drugs having the highest docking score of −10.60 and −7.51 kcal/mol after the screening of the drug library against MPXV cysteine proteinase (Fig. 3, Supplementary Table S2). Omadacycline is an oral aminomethylcycline antibiotic drug used for treating bacterial disease. This mainly treats pneumonia and acute skin infections such as acne^41^. Minocycline is an antibacterial antibiotic drug derived from tetracycline. They are active against active gram-negative and gram-positive bacteria^42^.

### Intermolecular interaction analysis

**Figure 4:**
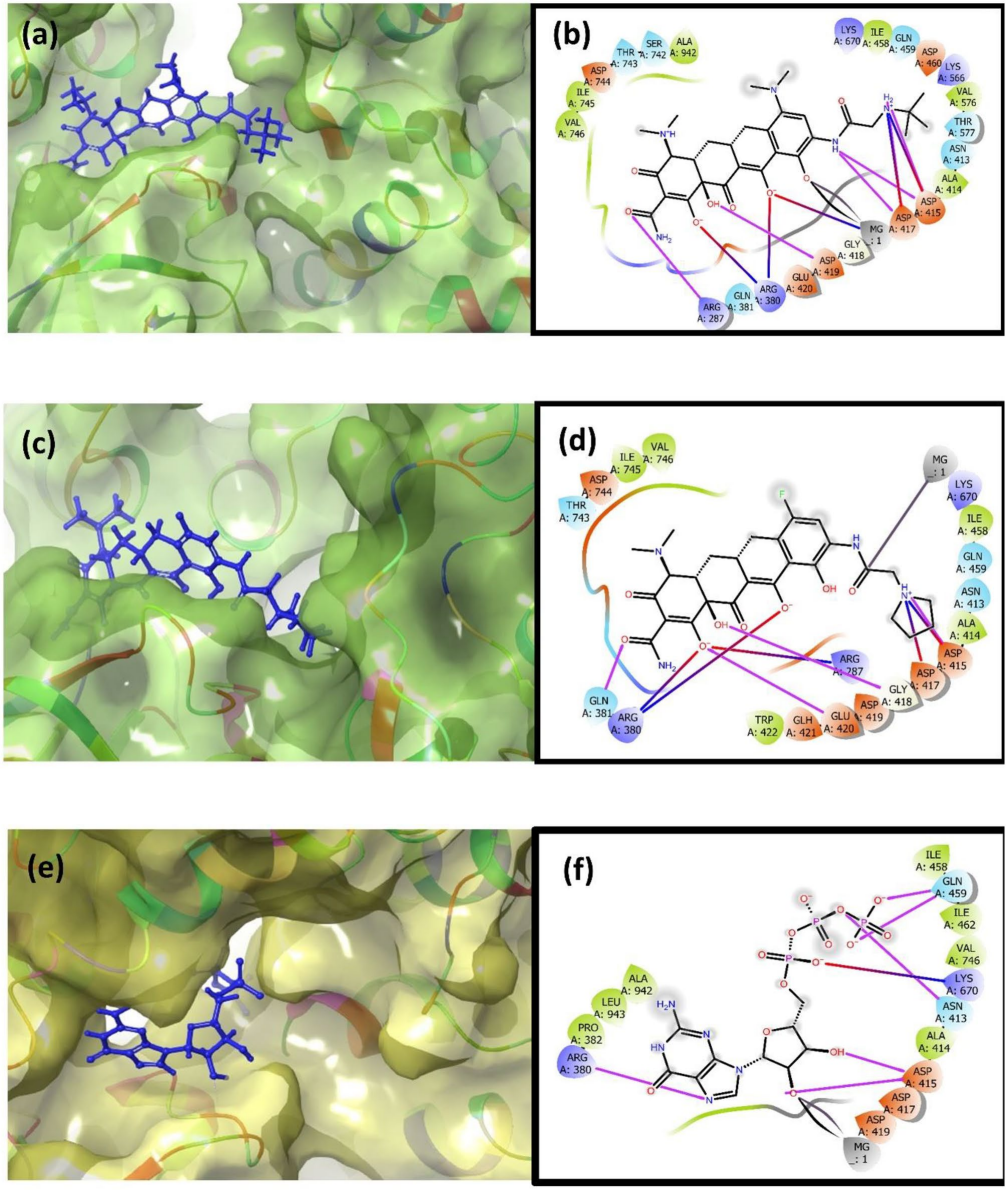
3D and 2D intermolecular interaction diagram of (**a**, **b**) Tigecycline, (**c**, **d**) Eravacycline and reference molecule (**e**, **f**) GTP. Herein, the pink line represents H-bond, red-violet line represents salt bridge, red colour petals represents negative charge, violet colour petal represents positive, green colour petals represents hydrophobic bond, and blue colour petal represents polar bond in the 2D interaction diagram of the complex.

The selected top two complexes and the MPXV protein reference molecules were studied to find the interaction between the protein–ligand complex. MPXV DdRp- Tigecycline complex significant hydrogen formation (6 H-bonds) with residues Asp^415^ (2), Asp^417^ (2), Asp^419^ and Arg^287^, whereas in DdRp-Eravacycline docked complex Asp^415^, Gly^418^, Glu^420^, and Gln^318^ residues are involved for hydrogen bond formation (4 H-bond). Moreover, when the intermolecular interaction of MPVX DdRp docked with reference compound GTP was observed, it showed a significant amount of hydrogen bond formation (6 H-bond). Apart from the hydrogen bond, these complexes exhibited salt bridge formations and hydrophobic, polar, positive and negative charge interactions. Based on this analysis, it can be concluded that tigecycline shows better binding stability among the two selected drugs with the DdRp of MPXV compared to reference molecule GTP (Table 1, Fig. 4).

**Table 1:** List of residues and type of intermolecular interaction involved when MPXV DdRp docked with selected drugs and *reference molecule.

S.no	Complex	H-bond	Hydrophobic	Polar	Salt bridge	Positive	Negative
1	DdRp-Tigecycline	Asp^415^ (2)	Ala^414^	Asn^413^	MG (metal ion)	Arg^287^	Asp^415^
		Asp^417^ (2)	Ile^458^	Gln^459^	Asp^415^	Arg^380^	Asp^417^
		Asp^419^	Val^576^	Thr^577^	Asp^417^	Lys^566^	Asp^419^
		Arg^287^	Ile^745^	Ser^742^	Arg^380^ (2)	Lys^670^	Glu^420^
			Val^746^	Thr^743^			Asp^460^
			Ala^942^				Asp^744^
2	DdRp-Eravacycline	Asp^415^	Ala^414^	Asn^413^	Asp^415^	Arg^287^	Asp^415^
		Gly^418^	Ile^458^	Gln^459^	Asp^417^	Arg^380^	Asp^417^
		Glu^420^	Trp^422^	Gln^318^	Arg^287^	Lys^670^	Asp^419^
		Gln^318^	Ile^745^	Thr^743^	Arg^380^ (2)		Glu^420^
			Val^746^				Glu^421^
							Asp^744^
3	*DdRp-GTP	Asn^413^	Pro^380^	Asn^413^	Lys^670^	Pro^380^	Asp^415^
		Asp^415^ (2)	Ala^414^	Gln^459^		Lys^670^	Asp^417^
		Gln^459^ (2)	Ile^458^				Asp^419^
		Arg^380^	Ile^462^				
			Val^746^				
			Ala^942^				
			Pro^943^

**Figure 5:**
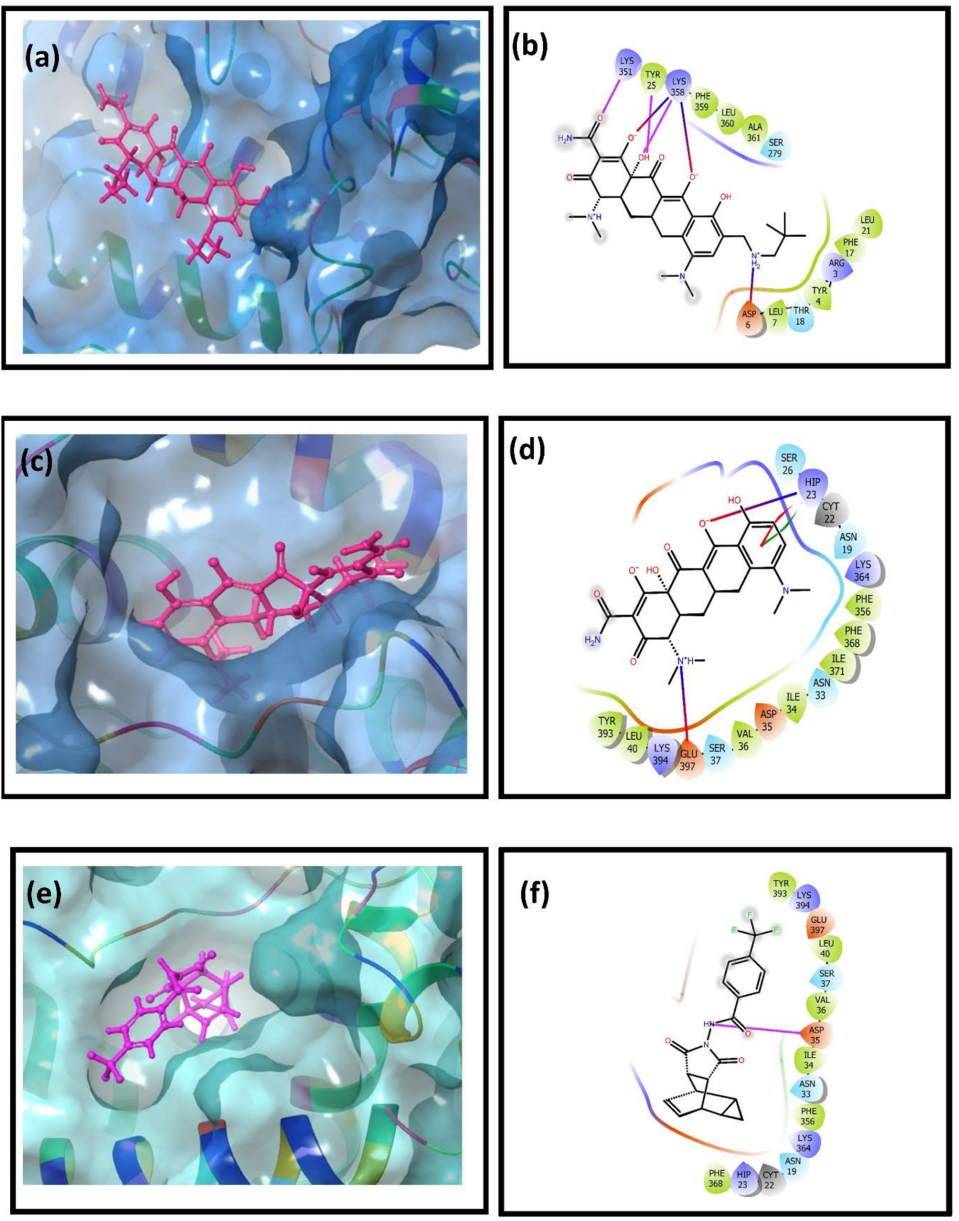
3D and 2D intermolecular interaction diagram of (**a**, **b**) Omadacycline, (**c**, **d**) Minocycline and reference molecule (**e**, **f**) Tecovirimat. Herein, the pink line represents H-bond, red-violet line represents salt bridge, red colour petals represents negative charge, violet colour petal represents positive, green colour petals represents hydrophobic bond, and blue colour petal represents polar bond in the 2D interaction diagram of the complex.

Similarly, when cysteine proteinase was docked with omadacycline drug, it was found that three residues, Tyr25, Lys351, and Lys358, are responsible for forming three hydrogen bonds in the complex. Interestingly, in the proteinase-minocycline complex, no hydrogen bonds were observed. However, the reference complex proteinase-tecovirimat displayed a single hydrogen bond formation with residue Asn^35^. Except for the reference complex, all the selected docked complexes show salt bridge formation. Moreover, hydrophobic interaction, polar bond, and positive and negative charge interaction were also observed in all the selected complexes, including the reference complex. The interaction analysis shows that omadacycline shows better binding stability towards MPXV cysteine proteinase than minocycline and reference molecule tecovirimat (Table 2, Fig. 5).

**Table 2:** List of residues and type of intermolecular interaction involved when MPXV proteinase docked with selected drugs and *reference molecule.

S.no	Complex	H-bond	Hydrophobic	Polar	Salt bridge	Positive	Negative
1	Proteinase-Omadacycline	Tyr^25^	Tyr^4^	Thr^18^	Asp^6^	Arg^3^	Asp^6^
		Lys^351^	Leu^7^	Ser^279^	Lys^358^	Lys^351^	
		Lys^358^	Phe^17^			Lys^358^	
			Leu^21^				
			Phe^359^				
			Leu^360^				
			Ala^361^				
2	Proteinase-Minocycline	–	Ile^34^	Asn^19^	His^23^	His^23^	Asp^35^
			Val^36^	Ser^26^	Glu^397^	Lys^364^	Glu^397^
			Leu^40^	Asn^33^		Lys^394^	
			Phe^356^	Ser^37^			
			Phe^368^				
			Ile^371^				
3	*Proteinase-Tecovirimat	Asn^35^	Ile^34^	Asn^19^	–	His^23^	Asp^35^
			Val^36^	Asn^33^		Lys^364^	Glu^397^
			Leu^40^	Ser^37^		Lys^394^	
			Phe^356^				
			Phe^368^				
			Phe^393^

### MD simulation analysis

**Figure 6:**
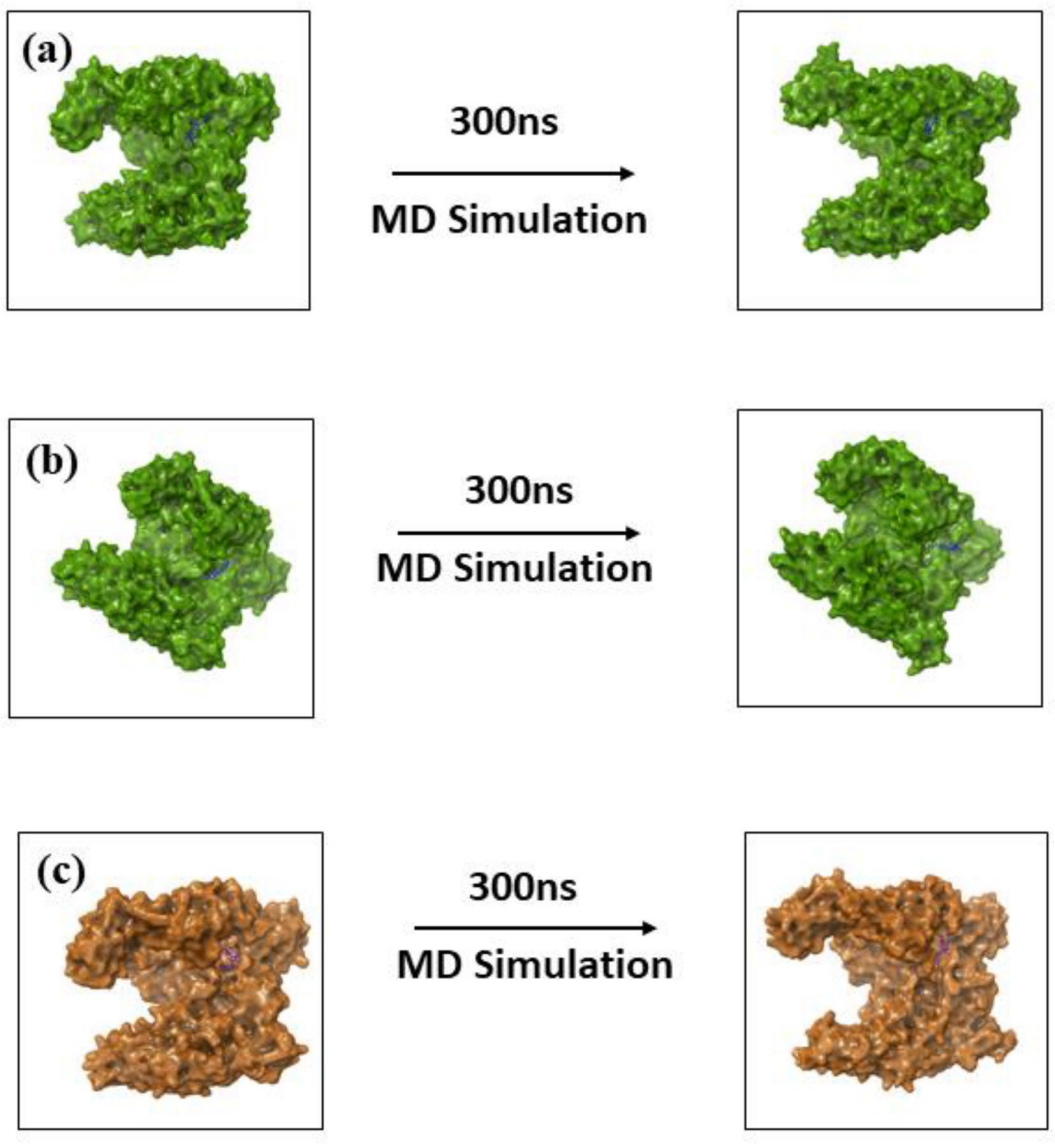
3D structure analysis of the first and last pose of (**a**) Tigecycline, (**b**) Eravacycline and reference molecule, (**c**) GTP extracted from the MD simulation trajectory of MPXV DdRp protein.

**Figure 7:**
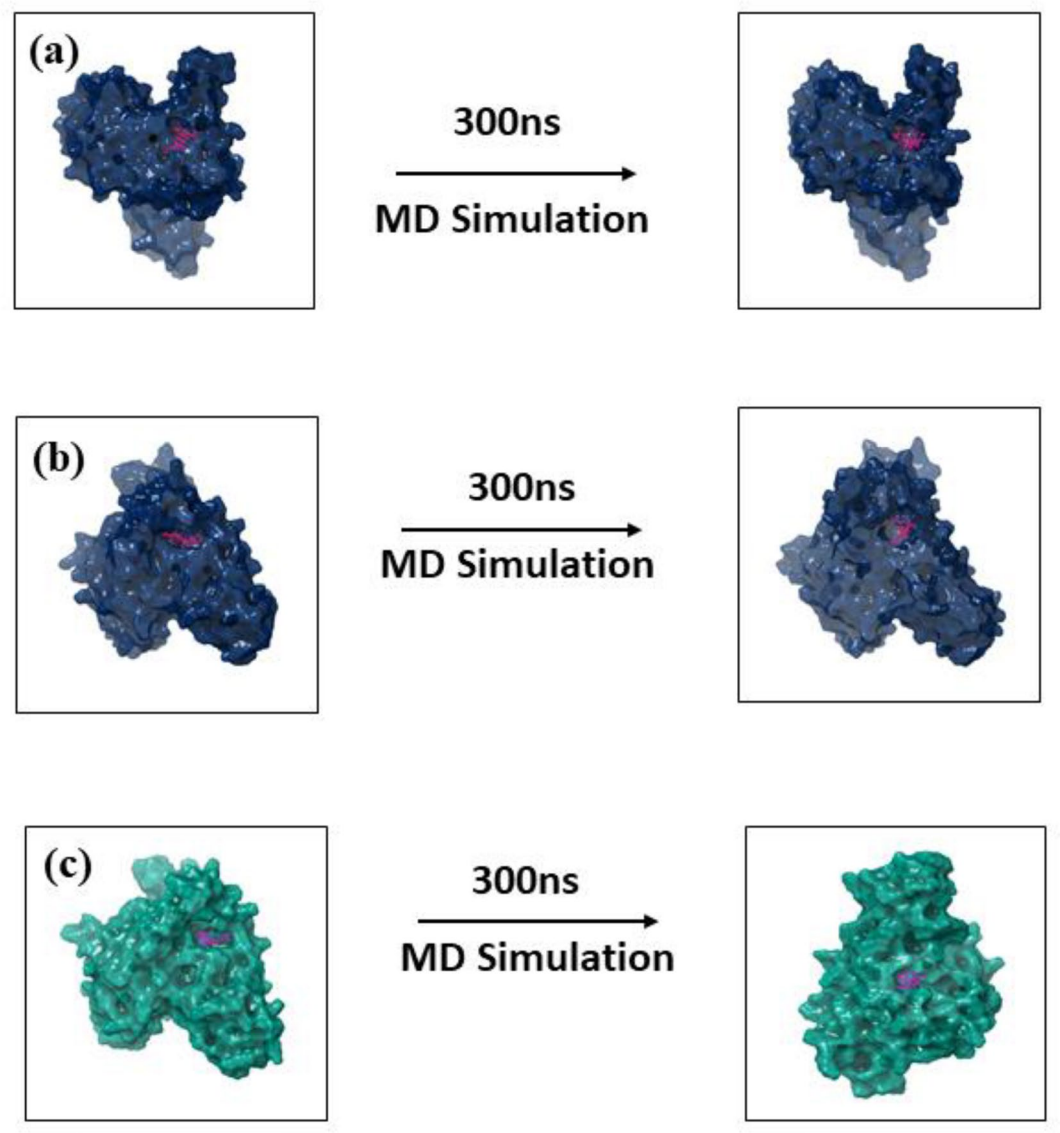
3D structure analysis of the first and last pose of (**a**) Omadacycline, (**b**) Minocycline and reference molecule, (**c**) Tecovirimat extracted from the MD simulation trajectory of MPXV proteinase protein.

Molecular dynamics simulation mainly focuses on calculating the natural dynamics of biomolecular structures on the different timescale in a solution. This also helps calculate each atom's fluid properties and movement in a system in a given set of times^43^. So, the selected protein–ligand complexes of both MPXV proteins were observed to understand the stability of the complex under 300 ns MD simulation. The 3D structure of the first and last post extracted from the MD simulation trajectory of both MPX proteins-ligand complex viz., DdRp and cysteine proteinase complexes were observed to understand the rotational and conformational changes between the initial pose and the final pose (Figs. 6, 7). The 3D structural analysis of the first and last poses of DdRp and cysteine proteinase complexes showed that both the target protein undergoes minor structural confirmations and the docked ligand molecules also exhibited structural rotation by the end of simulation period.

To understand the dynamic stability, the detailed analysis all the docked complex were carried out by calculating their Root mean square deviation (RMSD), and Root mean square fluctuation (RMSF). Also, protein–ligand contact mapping of these complexes from the MD simulation trajectories of both target proteins were also analyzed to find the interaction fractions of major residues during bond formation responsible for the stability of the protein–ligand complexes.

#### RMSD and RMSF analysis

**Figure 8:**
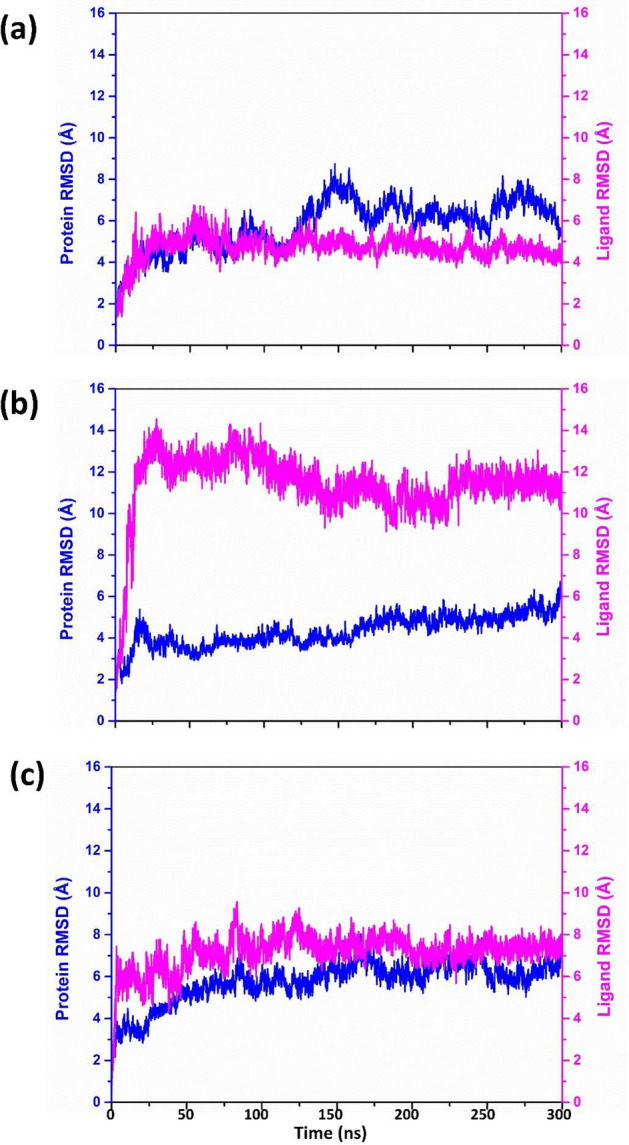
RMSD graph of (**a**) Tigecycline, (**b**) Eravacycline and reference molecule (**c**) GTP extracted from the 100 ns MD simulation trajectory of MPXV DdRp protein.

RMSD analysis calculates the conformational changes the protein and ligand undergo in the complex during the entire simulation period. During the protein RMSD analysis of the DdRp-tigecycline complex, the DdRp protein displayed a substantial deviation of 6 Å from 50 to 100 ns of simulation, which further increased to ≈ 9 Å at 150 ns, and by the end of 300 ns the protein deviated up to 8 Å. The protein RMSF analysis of this complex further confirmed this finding. The DdRp RMSF showed significant peaks having RMSF values of 6 Å between 200–400 residue index, followed by a 5 Å residual fluctuation between 600 and 800 residues, and finally two higher elevation (9 Å) between 1000 and 1200 residues. The fluctuations in the C-terminal and N-terminal ends of the protein are not considered. Based on the RMSD and RMSF analysis of the protein, it was found that the DdRp protein may have insignificant structural confirmational changes due to the binding of the drug molecule in the DdRp-tigecycline complex. However, the RMSD analysis of the tigecycline displayed a considerable deviation of 6 Å at 100 ns, but the ligand gradually attained stable equilibrium (4-5Å) after 100 ns till the end of simulation. RMSF analysis of tigecycline also exhibited low and stable fluctuation (< 3 Å) during the simulation. This concludes that the ligand molecule remained stable when attached to the binding site of the DdRp protein. In the case of DdRp- Eravacycline complex, the RMSD of the protein showed an insignificant deviation (> 5 Å) between 5 to 50 ns, which remained in stable equilibrium (5–6 Å) till the end of the simulation. Contrary to the protein, the ligand eravacycline displayed a very high deviation of 10 Å within the initial 20 ns and further increased up to 14 Å till 100 ns and the RMSD value fall to 12 Å by attaining equilibrium until the end of the simulation. Ligand RMSF analysis confirms this finding. The RMSF graph of the eravacycline showed multiple peaks of 6 Å value between 1 and 40 atoms of ligand molecule. On the basis of RMSD and RMSD analysis of the DdRp- Eravacycline complex, it was concluded that protein remain stable without any confirmation even though the drug exhibited significant deviation when docked with DdRp protein. However, the protein RMSD analysis of the reference DdRp-GTP complex showed an acceptable deviation (6 Å), by attaining a stable equilibrium state till the end of the simulation. The protein RMSF analysis also exhibited fluctuations of < 5 Å between 200 and 800 residual index and between 100 and 200 residual index the RMSF value of 6–7 Å were also noted during the trajectory analysis of the reference complex. The RMSD of the GTP showed a significant deviation between 6 and 8 Å till the end of the simulation, and the RMSF of all atoms of GTP showed 3 Å. Based on the RMSD and RMSF analysis of the DdRp-drug complex, it was found that, when tigecycline drug binds with the active site it induces confirmational change in the DdRp protein, whereas in case of Eravacycline, there is no confirmational changes are observed and the drug molecule remains bounded to the target protein, when compared with the reference compound GTP (Fig. 8, Supplementary Figures S1 and S2).

**Figure 9:**
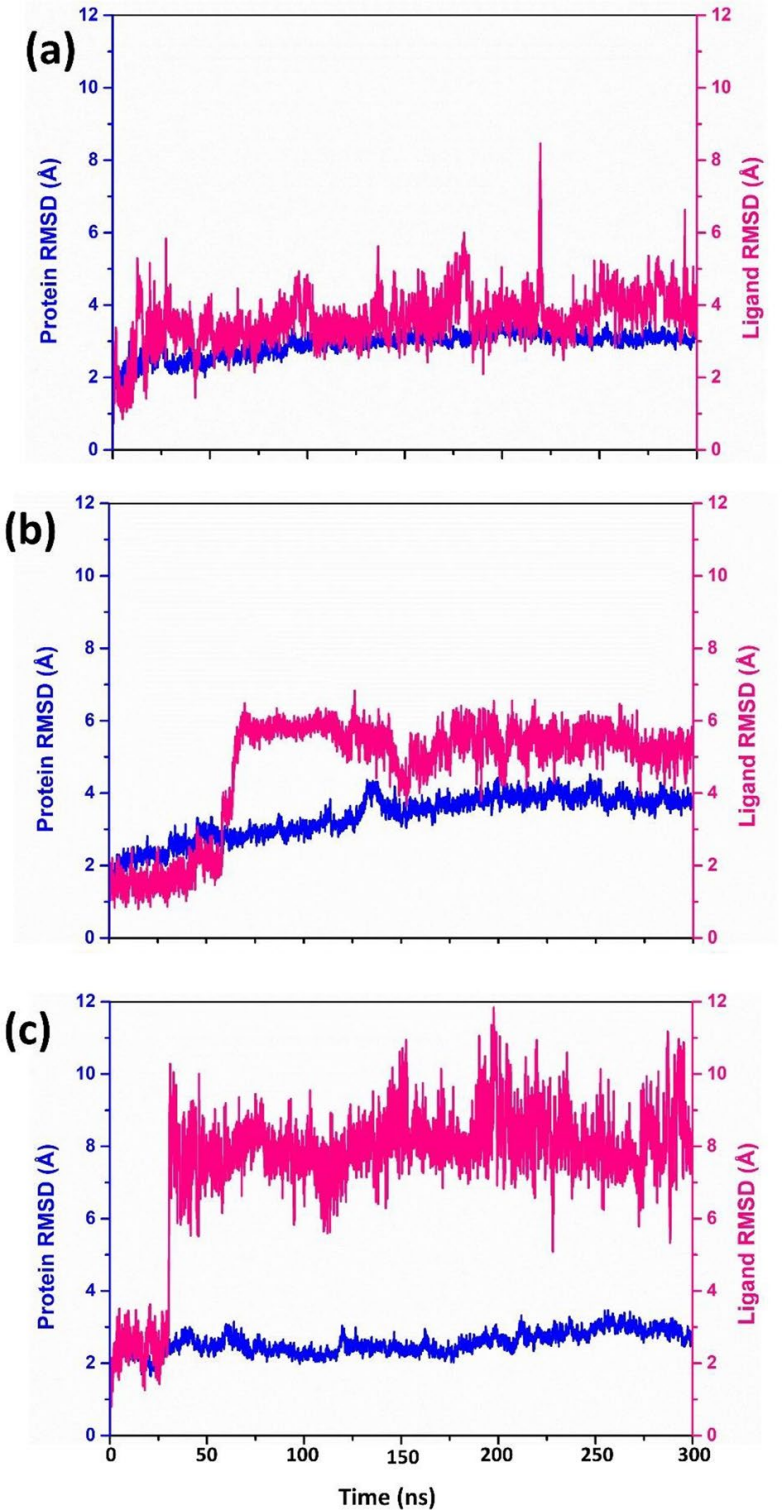
RMSD graph of (**a**) Omadacycline, (**b**) Minocycline and reference molecule (**c**) Tecovirimat extracted from the 100 ns MD simulation trajectory of MPXV cysteine proteinase protein.

Similarly, the RMSD graph of the proteinase in the proteinase-drug complex was also analysed. Based on the protein RMSD of these complexes, it was found that the MPXV proteinase remained in a stable state (< 4 Å) in all the protein–ligand complexes. This condition was also seen in the proteinase-tecovirimat reference complex. The protein RMSF analysis of the docked complexes also supports this information. The graph only shows a significant peak at residue number 150 and then drops to a stable state with an RMSF value below 2 Å. Likewise, during the RMSD analysis of the drug molecule in proteinase- omadacycline complex it was observed that the drug remain is stable sate (< 5 Å) with an insignificant fluctuation (8 Å) between 200 and 250 ns till the end of total simulation. The RMSF graph of omadacyclin displayed continuous peaks between 0 to 40 atoms, but the ligand RMSF value was in the acceptable range i.e., < 4 Å. The RMSD analysis of Minocycline showed stability (2 Å) till 60 ns and showed a substantial deviation of 6 Å after 60 ns and the gradually attains stable equilibrium till the end of the simulation. The RSMF analysis of minocycline supports this observation as the ligand shows acceptable fluctuations with an RMSF value of < 4 Å. However, the RMSD of reference molecule Tecovirimat remained stable during the initial 30 ns, followed by a significant rise of 10 Å, and then gradually attained the equilibrium stage (8 Å) at 100 ns simulation. At 200 ns the RMSD value of the reference molecule was further increased to12 Å and by the end of 300 ns simulation the RMSD value seen to in between 10 and 8 Å. The tecovirimat RMSF also exhibits two significant peaks with an RMSF value of > 4 Å, which gradually declined to 2 Å. Finally, it can be concluded, based on the RMSF and RMSD analysis of the proteinase-drug complex, that both the omadacycline and minocycline showed better stability compared to reference drug tecovirimit (Fig. 9, Supplementary Figures S3 and S4).

#### Protein–ligand contact mapping

**Figure 10:**
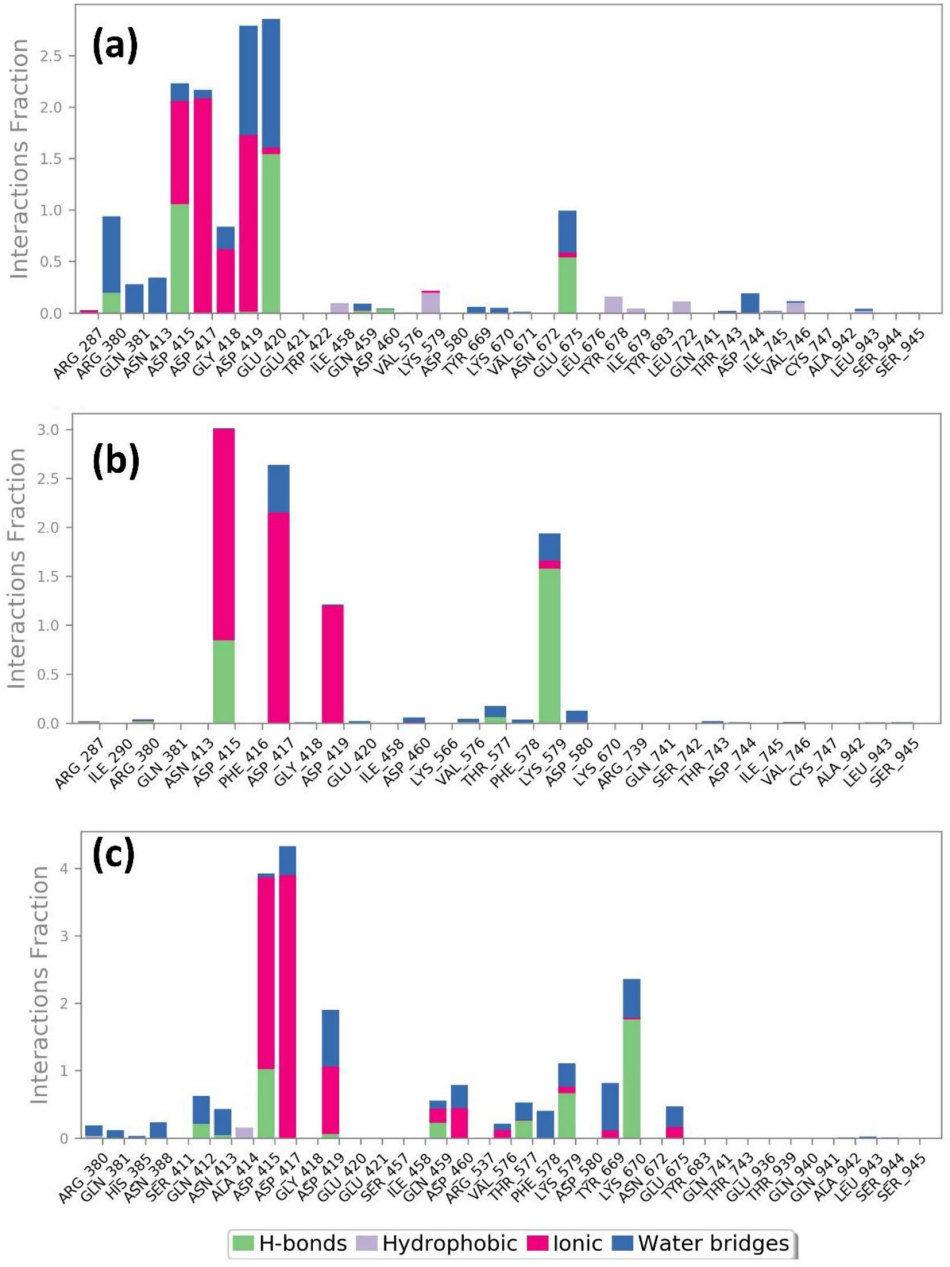
Protein–ligand interactions mapping for DdRp protein with selected antibiotic compounds over the 100 ns simulation (**a**) Tigecycline, (**b**) Eravacycline and reference compound (**c**) GTP.

To better understand the stability of mapping the protein–ligand interaction for the MPXV proteins is analysed. In the DdRp-tigecycline complex, Asp^415^ and Glu^420^ residues display hydrogen bonds for 100%, whereas Glu^675^ exhibited hydrogen bond for 50% of the total simulation time. Herein, Asp^415^, Asp^417^ and Asp^419^ showed ionic bond formation for 100% of the interaction fraction. Asp^419^ and Glu^420^ also exhibited water bridge formation for 100% of the total interaction fraction. Furthermore, the protein–ligand contact mapping of DdRp-Eravacycline complex showed ionic interaction with Asp^415^, Asp^417^ and Asp^419^ for 100% of 300 ns simulation. In this complex Asp^415^ and Lys^579^ exhibited hydrogen bond formation for 60% and 100% of the total simulation fraction, respectively. In the case of the reference complex also, Asp^415^, Asp^417^ and Asp^419^ displayed ionic bonds for 100% of the total interaction fraction, and Asp^415^ and Lys^670^ involved hydrogen bond formation for 100% until the end of the simulation. Some other residues in all these complexes also form hydrophobic bond formation. Ligand–protein contact schematic representation was also analysed, displaying significant residues involved in the interaction for more than 30% of the simulation time. During the schematic interaction diagram analysis, Asp^415^, Asp^417^, and Asp^419^ exhibited metallic bond formation with Magnesium ions in all three DdRp-ligand complexes. Also, in the reference ligand contact diagram, Sodium ions were responsible for metallic bond formation. Apart from the metal coordinate bond, these residues are also involved in hydrogen bond formation for more than 30% of the simulation time (Fig. 10, Supplementary Figure S5).

**Figure 11:**
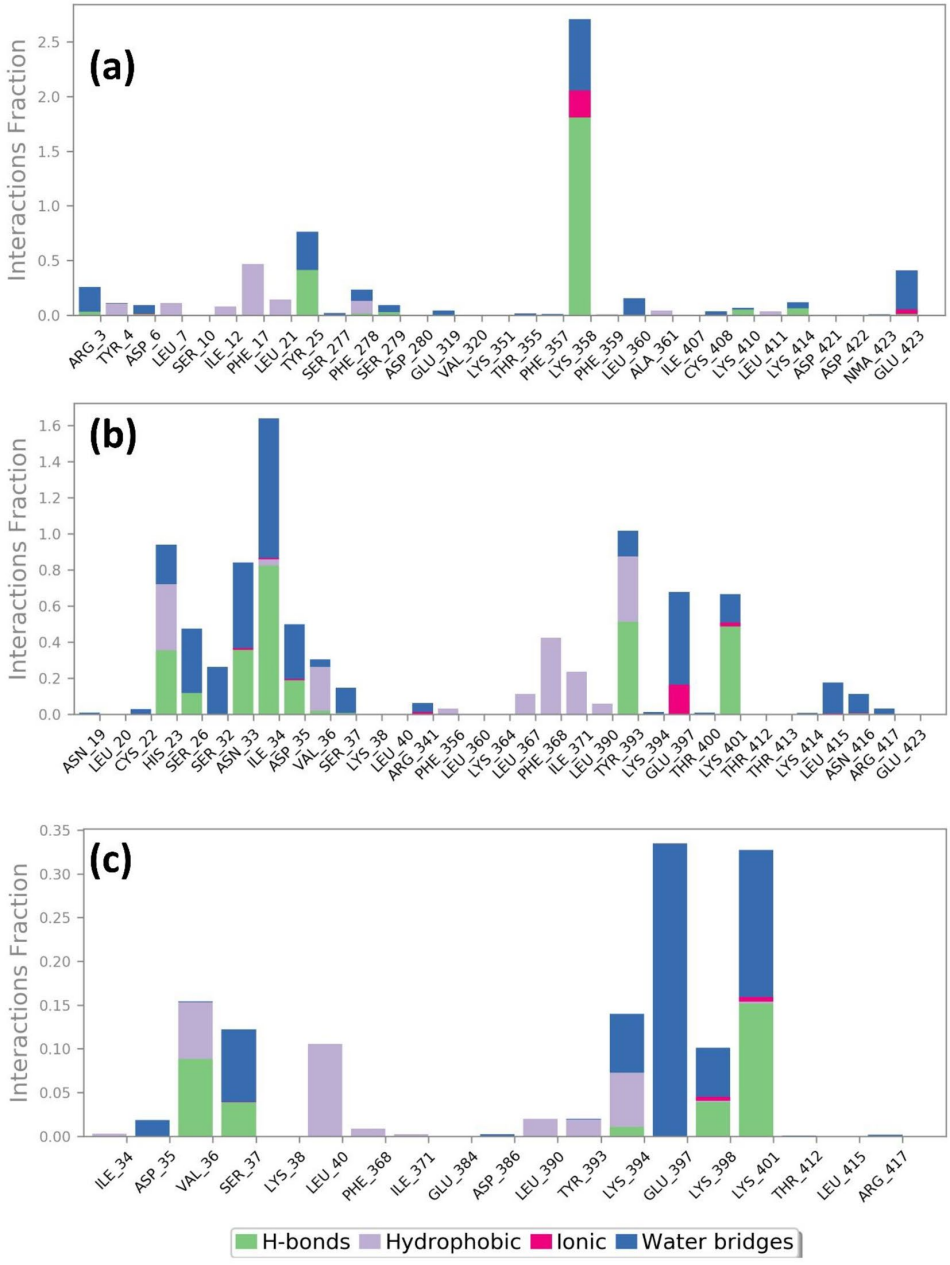
Protein–ligand interactions mapping for cysteine proteinase protein with selected antibiotic compounds over the 100 ns simulation (**a**) Omadacycline, (**b**) Minocycline and reference compound, (**c**) tecovirimat.

Similarly, the protein–ligand contact mapping of proteinase-omadacycline complex exhibited hydrogen bond formation with Tyr^25^ and Lys^358^ residues for 40% and 100% of the total simulation time, respectively, along with water bridge formation for 50% of the interaction time. Herein, the Phe^17^ residue displays hydrophobic interaction for 40% of the total interaction fraction. In the proteinase-minocycline complex His^23^ (35%), Asn^33^ (40%), Ile^34^ (80%), Tyr^393^ (60%) and Lys^401^ (60%) exhibited hydrogen bond formation of the total interaction fraction. In this complex water bridges were also formed with the residues Ser^26^ (30%), Ser^32^ (30%), Asn^33^ (40%), and Ile^34^ (100%) during the MD simulation. Also in this complex, His^23^ and Phe^368^ residues participates in hydrophobic and ionic bond for 40% of the 300 ns simulation time. Moreover, the reference complex exhibited hydrogen bond formation for 10% and 15% of the total simulation time with residues Val^36^ and Lys^401^. Glu^397^ and Lys^401^ displays water bridge formation for 35% and 25% of the total interaction fraction. Hydrophobic bonds were also formed with residue Leu^40^ for 10% of the simulation time. The schematic diagram of the ligand contact also shows the involvement of active site residues that make contact with the ligand for more than 30% of the simulation time (Fig. 11, Supplementary Fig. S6).

## Free binding energy analysis

**Figure 12:**
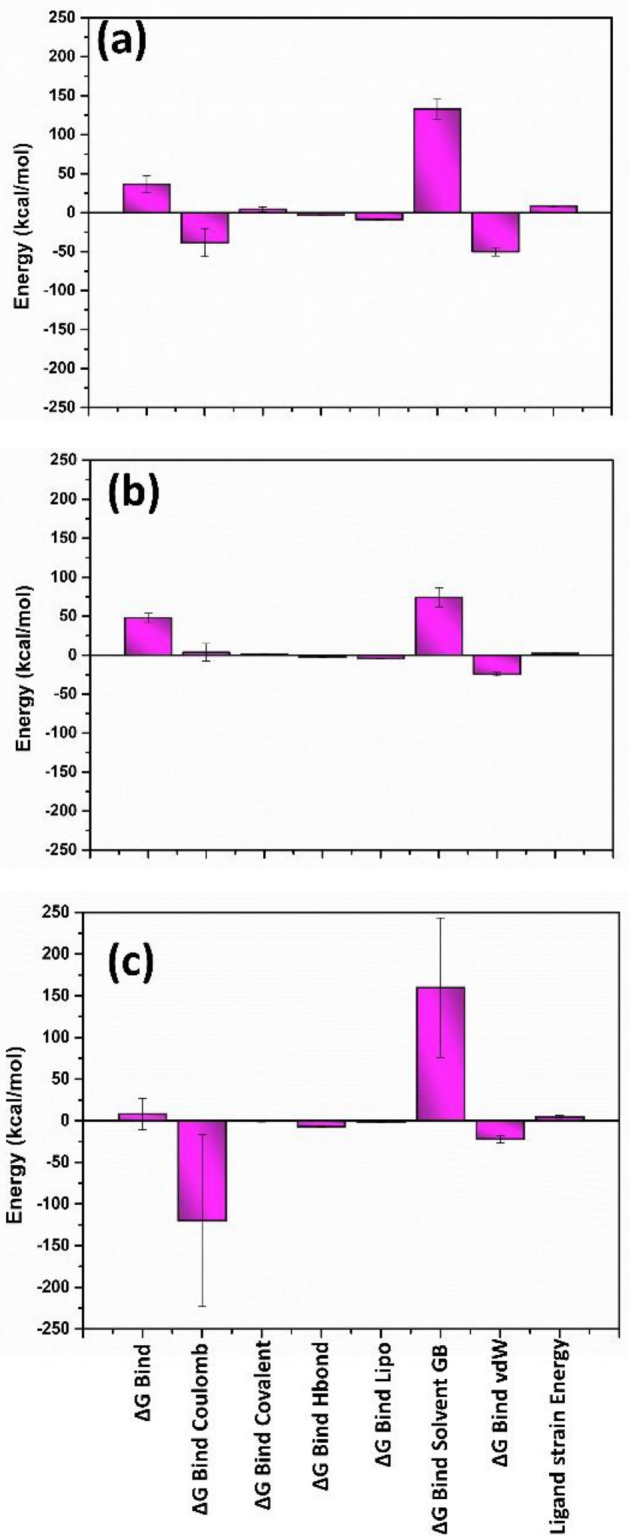
Free binding energy graph plotted for (**a**) Tigecycline, (**b**) Eravacycline and reference compound (**c**) GTP.

The free binding energy of both the selected ligands of the viral protein was calculated from the trajectories during the last 10 ns to estimate the binding affinity of the selected drugs at the binding site of the protein. The energy components such as ΔG_Bind,_ ∆G _Bind Coulomb_, ΔG_Bind-covalent_, ΔG_Bind-Hbond_, ΔG_Bind-Lipo_, ΔG_Bind-SolvGB,_ ΔG_Bind-vdW_ and ligand strain energy were calculated and plotted using the MM/GBSA calculation method for the top hit compounds of each viral protein and reference compounds. In the DdRp-ligand complex, ∆G _Bind Coulomb_ and ΔG_Bind-vdW_ contribute the most binding of the ligand with the protein, whereas ΔG_Bind_ and ΔG_Bind-SolvGB_ show a minor contribution as they show positive binding energy. (Fig. 12, Supplementary Table S3).

**Figure 13:**
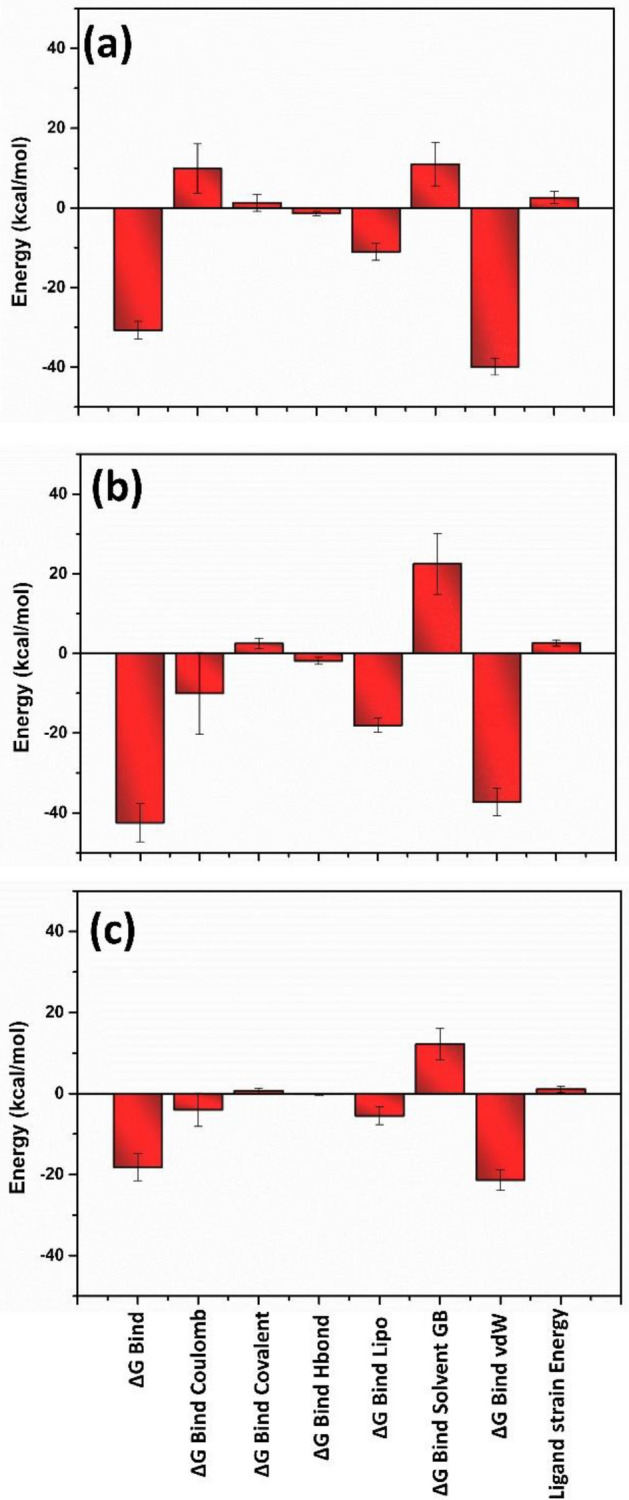
Free binding energy graph plotted for (**a**) Omadacycline, (**b**) Minocycline and reference compound (**c**) tecovirimat.

Similarly, in the proteinase-ligand complex, ΔG_Bind_ and ΔG_Bind-vdW_ energy significantly contribute to ligand binding affinity with proteinase. Herein minocycline showed the highest ΔG_Bind_ energy value compared to omadacycline and reference molecule. The ΔG_Bind-covalent_ and ΔG_Bind-SolvGB_ showed the minor contribution in the binding affinity of the ligand to the binding site of the proteinase protein (Fig. 13, Supplementary Table S4).

## Principal component analysis (PCA)

**Figure 14:**
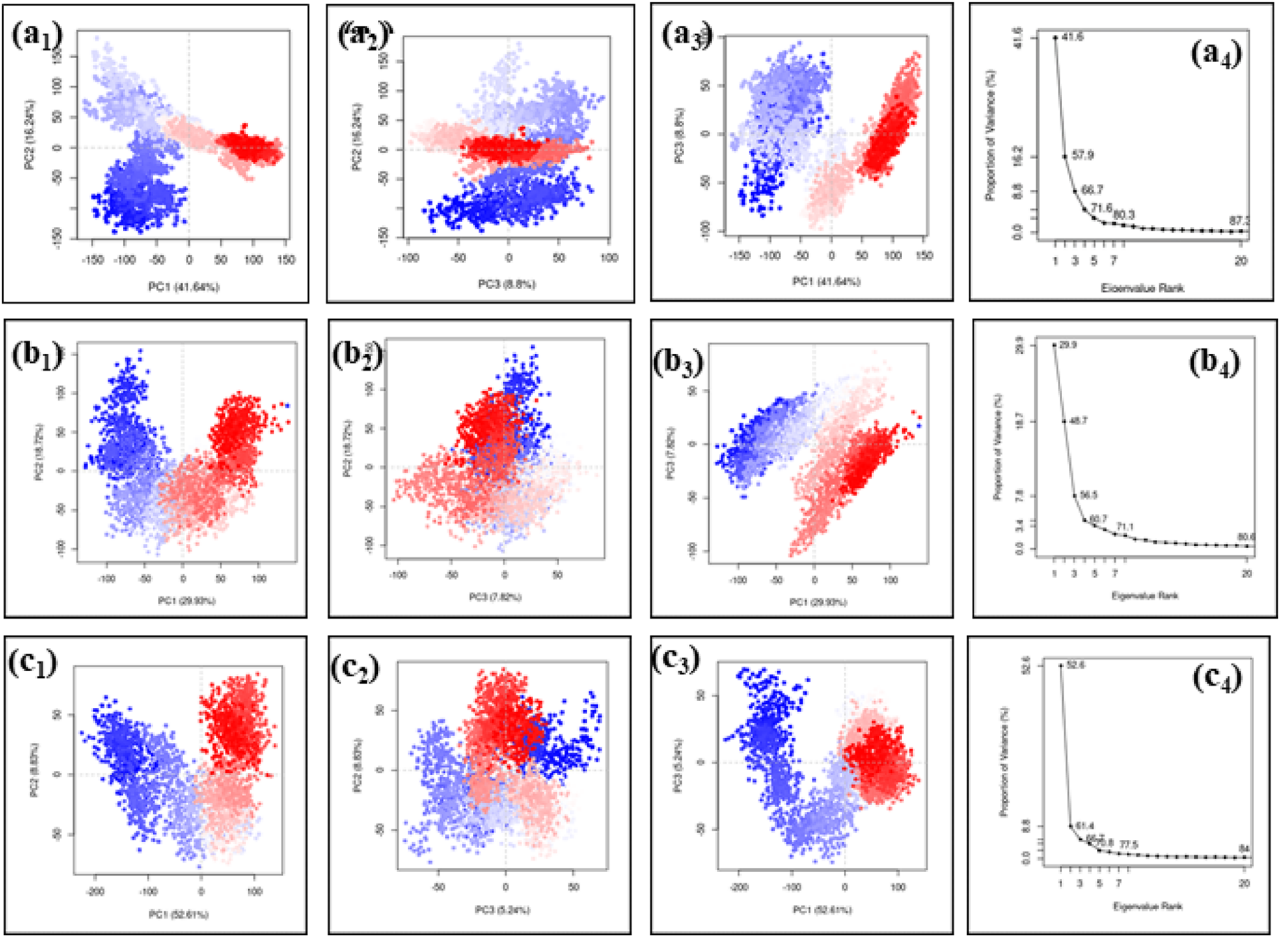
PCA analysis of the (**a**) Tigecycline, (**b**) Eravacycline and reference compound (**c**) GTP molecule docked with DdRp complex.

Principal Component Analysis (PCA) serves as a pivotal method for dimensionality reduction in complex datasets, with a core objective of preserving essential information. Through this technique, a triad of linear combinations, referred to as principal components, is extracted from the data. These principal components are meticulously crafted to encapsulate the most significant variability present within the dataset, thus ensuring a comprehensive representation of the data's inherent patterns and trends. Three essential components or principal components named PC1, PC2 and PC3 were retrived from the MD simulation trajectories having maximum amount of data are taken for PCA analysis. The PCA analysis of DdRp- Tigecycline complex exhibited a total of 66.68% during the summation of summation of three principal component (PC1 + PC2 + PC3). The cluster analysis of this complex showed there is no overlapping of three colours (red, white and blue) and finally the scree plot also exhibited a partial slope formation. Based on these observations, it was concluded that the protein may have undergone certain structural confirmation change due to the binding of the ligand molecule. Similarly in case of DdRp- Eravacycline complex, the total percentage obtained by calculating the three principal components was 56.47%. In this complex also the clusters are partially overlapped and scattered and in the scree plot shows fall of eigen values in steep pattern. In this also complex, it was protein tends be flexible in nature due to the binding of ligand. Similarly, in the reference complex, DdRp-GTP also, the total PCA value was 66.68 percentage similar to the tigecycline complex. And the clusters are scattered and a sharp fall was seen in the scree plot, stating the fact the protein molecule have undergone confirmational changes during the simulation analysis. Based on the observation made during the PCA analysis of DdRP-drug complexes it was concluded that the protein may undergo confirmation changes due to the binding of drug molecule similar to the reference molecule (Fig. 14).

**Figure 15:**
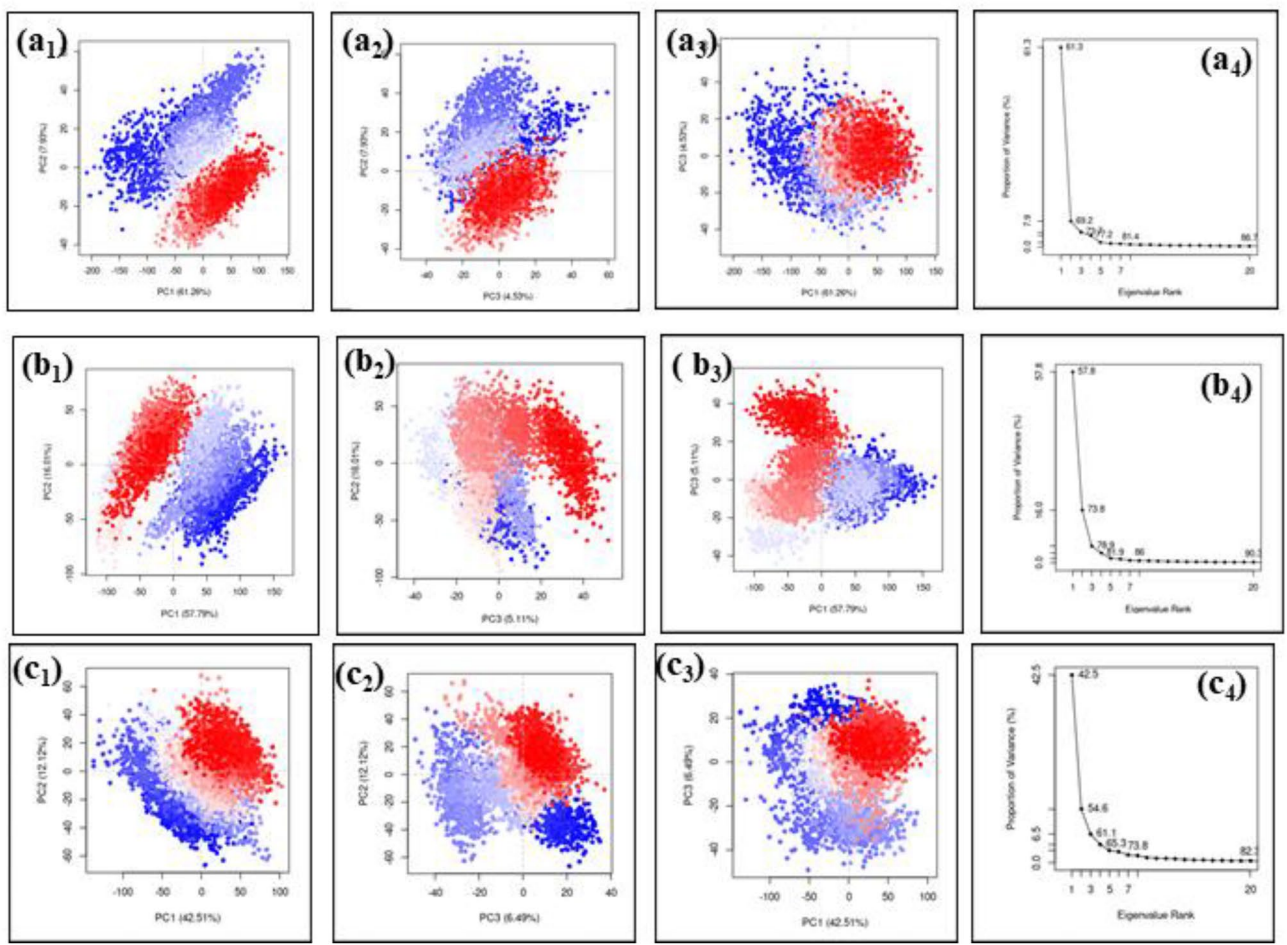
PCA analysis of (**a**) Omadacycline, (**b**) Minocycline and reference compound (**c**) tecovirimat docked with cysteine proteinase.

Likewise, the PCA analysis of the cysteine proteinase-drug complexes were also observed. Herein, the cysteine-proteinase-omadacycline complex, exhibited a total value of 73.72% during the total calculation of the three essential components. Also, the cluster analysis of this complex exhibited overlapping of colours present in clusters and the scree plot shows a sharp elbow shaped slope formation. These observations explains that the protein remain in stable state with minor to no confirmational changes. Similarly, in the Minocycline complex also, the cluster overlapping can observe, having total PCA value of 78.91% and the scree plot also shows sharp slope formations. This also states that chances of protein undergoing confirmational changes are less. Finally, during the PCA analysis of the reference molecule the clusters are overlapped. The total PCA value obtained in this complex, was 61.12% lesser than selected drug molecule. The scree plot showed a slight cure by the end of plot, stating that here also the protein molecule remains in rigid state with minor to no confirmational changes. Overall, it can be concluded that the cysteine proteinase does undergoes any kind of confirmational changes due to the binding of drug molecules by may tend to inhibit the function of the protein (Fig. 15).

## Conclusion

Serological and molecular testing is currently used to detect MPXV infection, a recently re-emerged pathogen. MPXV can spread via zoonotic reservoirs from animal to human and then from human to human. DdRp and viral core cysteine proteinase of MPXV play a significant role in viral replication cycle. Therefore, these two proteins are primary targets for drug development. Herein, the 3D structure of both proteins were modelled using the homology modelling technique due to the unavailability of experimentally proven 3D structures. The protein models of both proteins, DdRp and cysteine proteinase, were screened against the 16 tetracycline groups of antibiotics. Tigecycline and Evaracycline showed the highest docking score when screened against DdRp protein. MD simulation analysis of these compounds showed that tigecycline has better binding stability towards DdRp than tetracycline and reference compound GTP. The RMSD, RMSF and protein–ligand contact mapping analysis of each selected complex proved this. Similarly, while screening drugs against proteinase, the top two compounds, omadacycline and minocycline, displayed the highest docking score. The MD simulation analysis of these complexes showed that omadacycline has a better binding affinity towards proteinase. These two drug molecules show potential inhibitory properties against MPXV DdRp and proteinase protein.

### Supplementary Information

Supplementary Information.

## Data Availability

The datasets generated and/or analysed during the current study are available upon request from the corresponding author.
