# Supplementary material for: A multi-targeted computational drug discovery approach for repurposing tetracyclines against monkeypox virus
Source: Sci Rep. 2023 Sep 4;13:14570. doi: 10.1038/s41598-023-41820-z (PMC10477205; doi:10.1038/s41598-023-41820-z)
Supplement: Supplementary file 1 — Supplementary Information. [file 41598_2023_41820_MOESM1_ESM.docx]

Supplementary material

Result and Discussion

1. High-throughput virtual screening

Supplementary Table 1- HTVS result of tetracycline drug library screened again DNA dependent RNA polymerase

| PUBCHEM_COMPOUND_CID | Docking score (kcal/mol) |
| --- | --- |
| 54686904 | -8.888 |
| 54726192 | -7.871 |
| 54697325 | -7.868 |
| 54726192 | -7.789 |
| 54680675 | -7.248 |
| 54726192 | -7.24 |
| 54681908 | -7.151 |
| 54686904 | -7.094 |
| 54697325 | -7.05 |
| 54707177 | -7.01 |
| 54707177 | -6.976 |
| 54697325 | -6.915 |
| 54707177 | -6.658 |
| 54686187 | -6.617 |
| 54686904 | -6.426 |
| 54726192 | -6.073 |
| 54675785 | -6.015 |
| 54686187 | -5.971 |
| 54680690 | -5.822 |
| 54675783 | -5.666 |
| 54686187 | -5.587 |
| 54686904 | -5.579 |
| 54681908 | -5.422 |
| 54675776 | -5.421 |
| 54676539 | -5.408 |
| 54680675 | -5.061 |
| 54681908 | -5.014 |
| 54686187 | -4.947 |
| 54675785 | -4.875 |
| 54682938 | -4.796 |
| 54680675 | -4.703 |
| 54682938 | -4.679 |
| 54680675 | -4.646 |
| 54682938 | -4.555 |
| 54707177 | -4.528 |
| 54680690 | -4.46 |
| 54686187 | -4.424 |
| 54675776 | -4.403 |
| 54726192 | -4.384 |
| 54675777 | -4.374 |
| 54707177 | -4.329 |
| 54675777 | -4.204 |
| 54675779 | -4.188 |
| 54686187 | -4.072 |
| 54675783 | -4.065 |
| 54726192 | -3.847 |
| 54682938 | -3.778 |
| 54682938 | -3.593 |
| 54675783 | -3.588 |
| 54681908 | -3.579 |
| 54697325 | -3.563 |
| 54686904 | -3.464 |
| 54686904 | -3.379 |
| 54681908 | -3.305 |
| 54675776 | -3.219 |
| 54671203 | -3.182 |
| 54681908 | -3.117 |
| 54680690 | -2.941 |
| 54726192 | -2.928 |
| 54675777 | -2.889 |
| 54682938 | -2.862 |
| 54676539 | -2.833 |
| 54675777 | -2.729 |
| 54675776 | -2.674 |
| 54697325 | -2.626 |
| 54671203 | -2.204 |
| 54676539 | -2.012 |
| 54675783 | -2.003 |
| 54680690 | -1.931 |
| 54707177 | -1.735 |
| 54707177 | -1.216 |
| 54675779 | -1.198 |
| 54697325 | -1.078 |
| 54686187 | -0.72 |
| 54681908 | -0.715 |
| 54686187 | -0.621 |
| 54681908 | -0.198 |

Supplementary Table 2- HTVS result of tetracycline drug library screened again proteinase

| PUBCHEM_COMPOUND_CID | Docking score (kcal/mol) |
| --- | --- |
| 54697325 | -10.600 |
| 54675783 | -7.513 |
| 54726192 | -7.233 |
| 54726192 | -6.796 |
| 54675779 | -6.293 |
| 54675776 | -5.644 |
| 54671203 | -5.522 |
| 54680690 | -5.312 |
| 54682938 | -5.306 |
| 54686187 | -4.990 |
| 54686187 | -4.950 |
| 54675779 | -4.909 |
| 54675777 | -4.841 |
| 54675776 | -4.788 |
| 54676539 | -4.518 |
| 54697325 | -4.123 |
| 54686187 | -4.050 |
| 54681908 | -4.020 |
| 54686904 | -3.951 |
| 54675777 | -3.866 |
| 54707177 | -3.832 |
| 54675785 | -3.816 |
| 54707177 | -3.808 |
| 54707177 | -3.651 |
| 54697325 | -3.620 |
| 54680690 | -3.568 |
| 54676539 | -3.557 |
| 54671203 | -3.530 |
| 54675776 | -3.496 |
| 54726192 | -3.420 |
| 54675783 | -3.152 |
| 54726192 | -3.059 |
| 54675783 | -3.059 |
| 54680675 | -3.046 |
| 54686187 | -3.014 |
| 54681908 | -2.998 |
| 54680690 | -2.989 |
| 54682938 | -2.941 |
| 54726192 | -2.726 |
| 54707177 | -2.709 |
| 54682938 | -2.707 |
| 54675777 | -2.703 |
| 54686904 | -2.384 |
| 54686904 | -2.337 |
| 54680690 | -2.220 |
| 54686904 | -2.074 |
| 54680675 | -2.030 |
| 54671203 | -1.870 |
| 54681908 | -1.832 |
| 54675785 | -1.761 |
| 54680675 | -1.699 |
| 54697325 | -1.670 |
| 54707177 | -1.498 |
| 54681908 | -1.493 |
| 54686187 | -1.462 |
| 54686904 | -1.405 |
| 54675777 | -1.377 |
| 54681908 | -1.286 |
| 54675776 | -1.142 |
| 54686187 | -1.121 |
| 54675783 | -1.017 |
| 54681908 | -0.740 |
| 54686904 | -0.645 |
| 54680690 | -0.566 |
| 54682938 | -0.496 |
| 54680675 | -0.460 |
| 54682938 | -0.356 |
| 54675776 | -0.246 |
| 54686187 | -0.117 |
| 54675777 | -0.049 |

2. MD simulation analysis


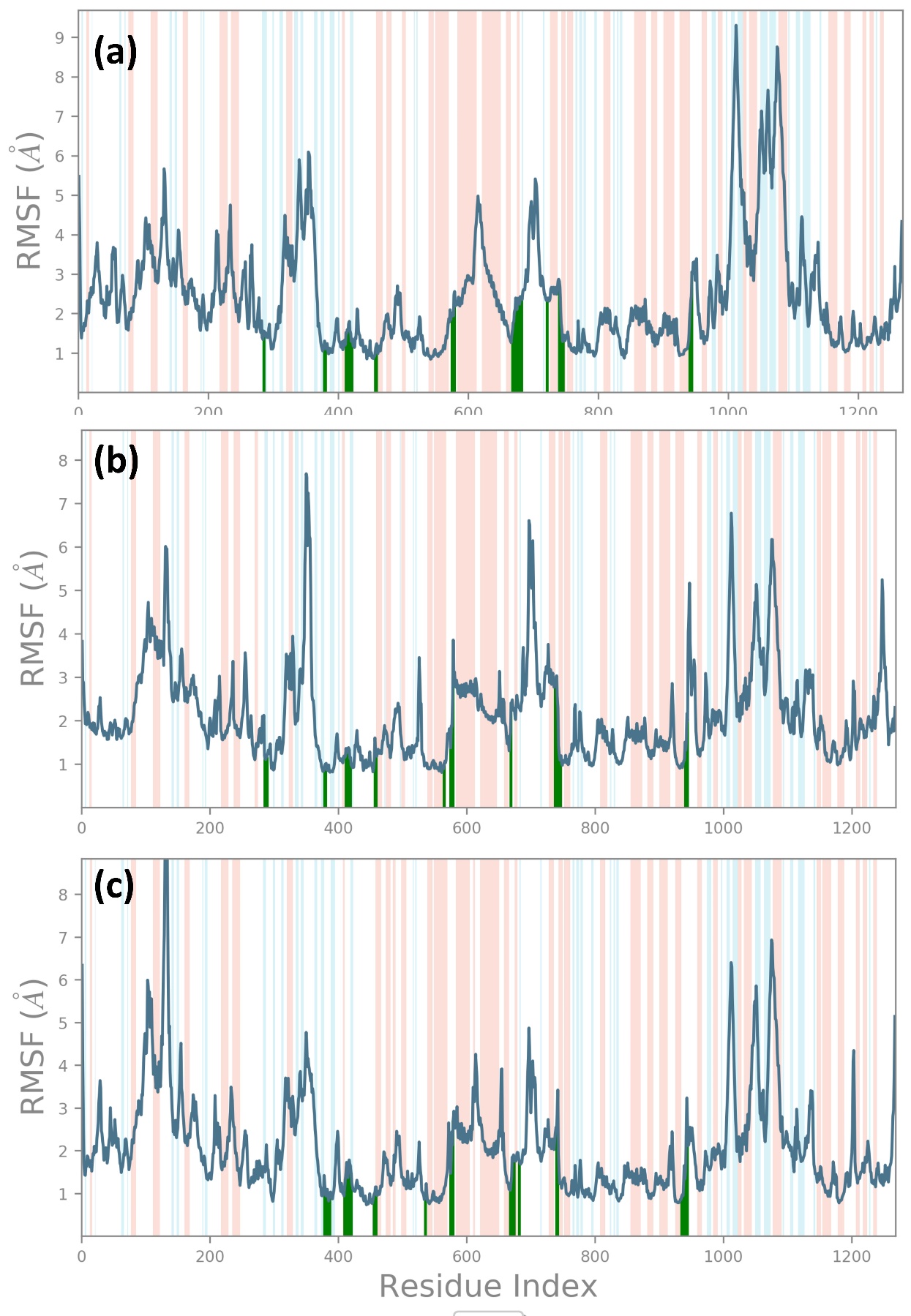


Supplementary Figure 1- Protein RMSF graph of (a) Tigecycline, (b) Eravacycline and reference molecule (c) GTP extracted from the 100ns MD simulation trajectory of MPXV DdRp protein


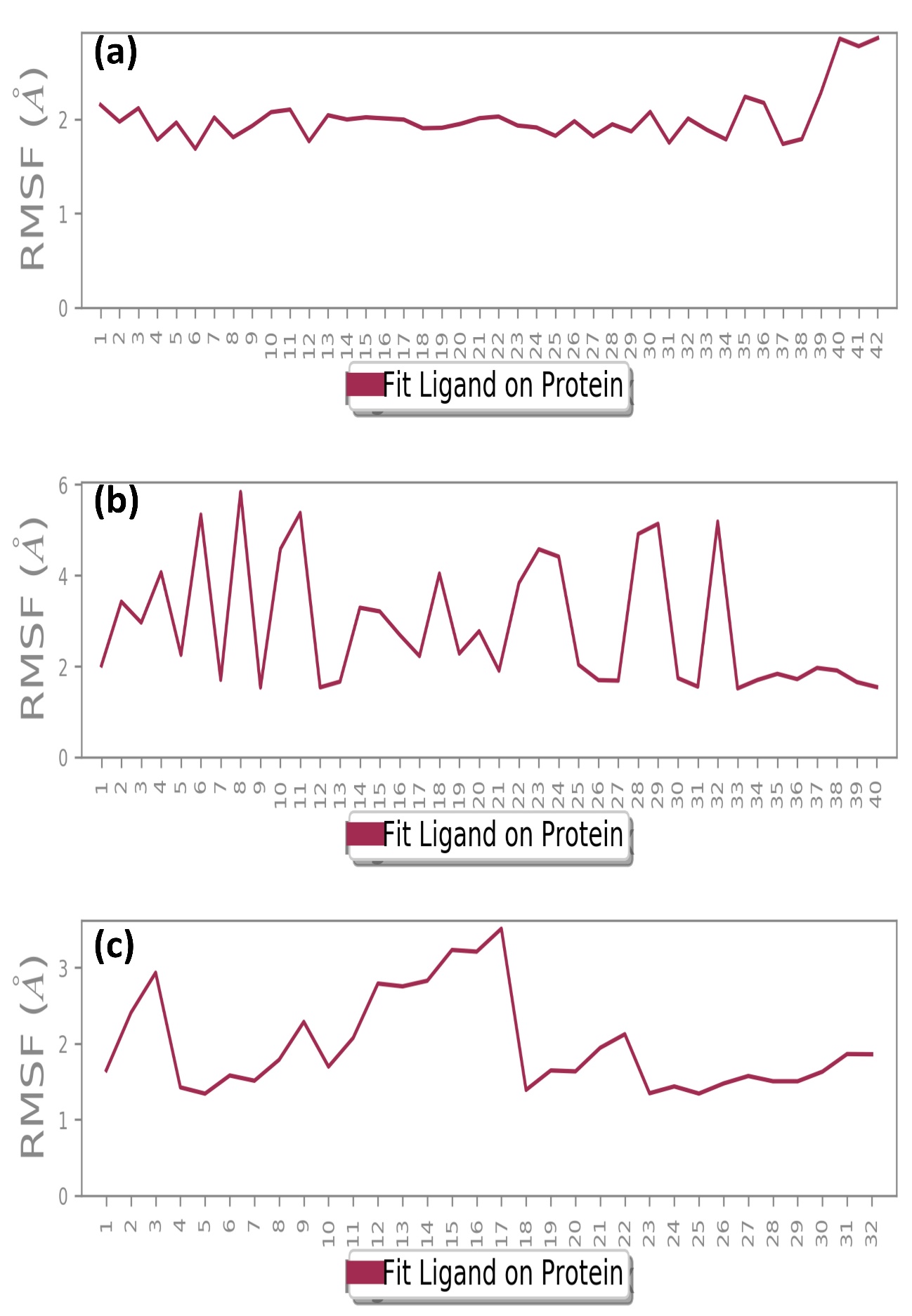


Supplementary Figure 2- Ligand RMSF graph of (a) Tigecycline, (b) Eravacycline and reference molecule (c) GTP extracted from the 100ns MD simulation trajectory of MPXV DdRp protein.


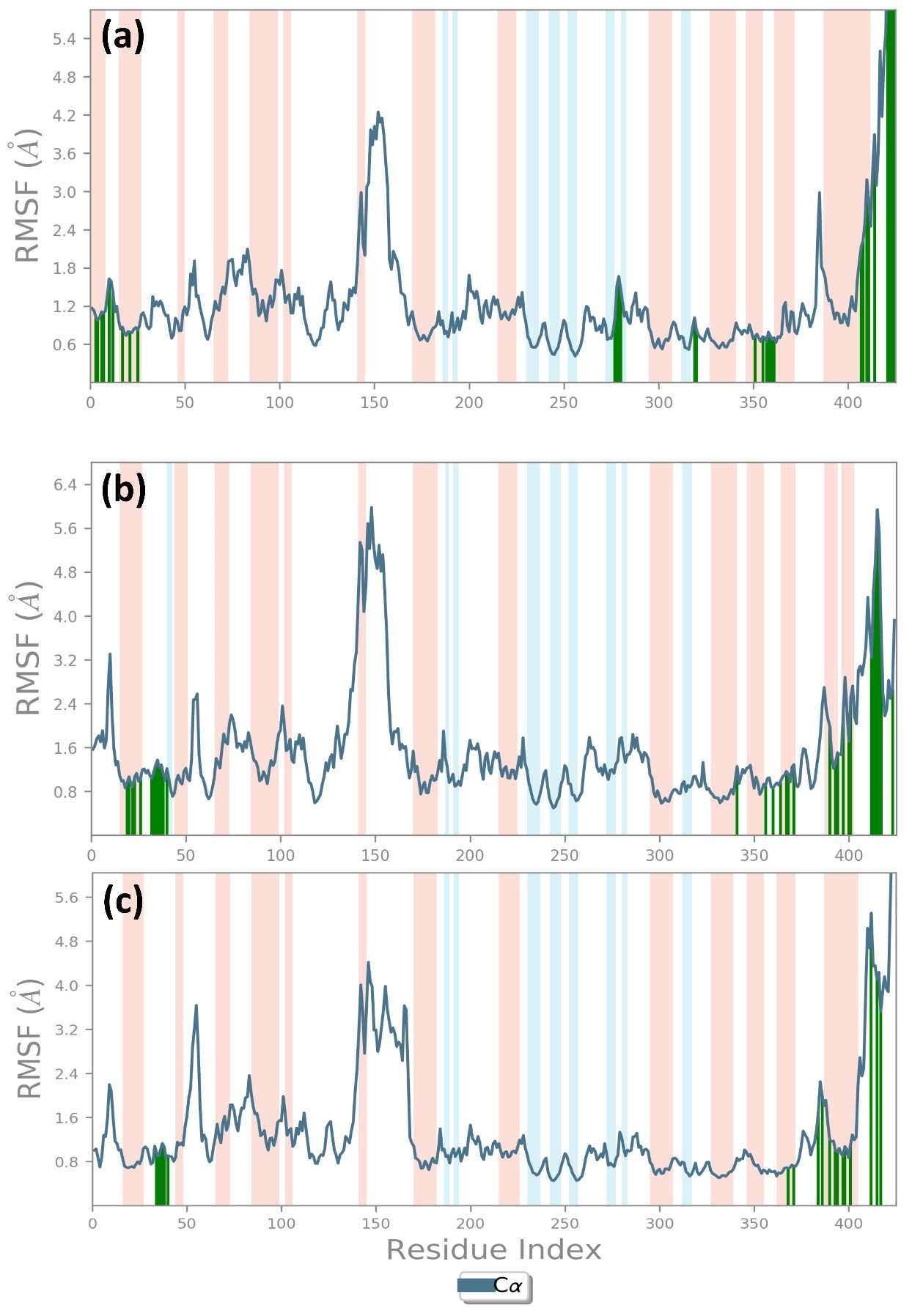


Supplementary Figure 3- Protein RMSF graph of (a) Omadacycline, (b) Minocycline and reference molecule (c) Tecovirimat extracted from the 100ns MD simulation trajectory of MPXV cysteine proteinase protein.


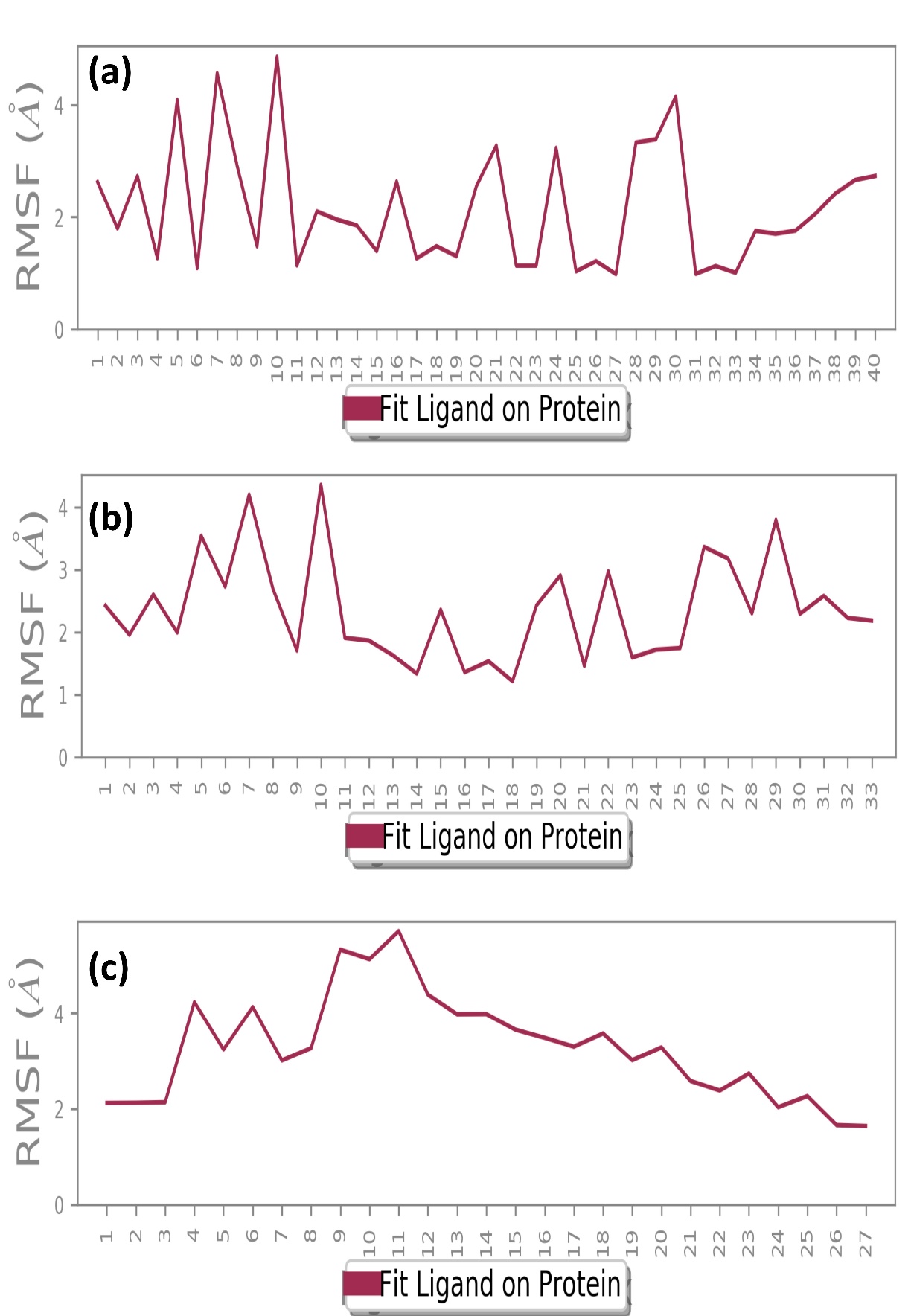


Supplementary Figure 4- Ligand RMSF graph of (a) Omadacycline, (b) Minocycline and reference molecule (c) Tecovirimat extracted from the 100ns MD simulation trajectory of MPXV cysteine proteinase protein.


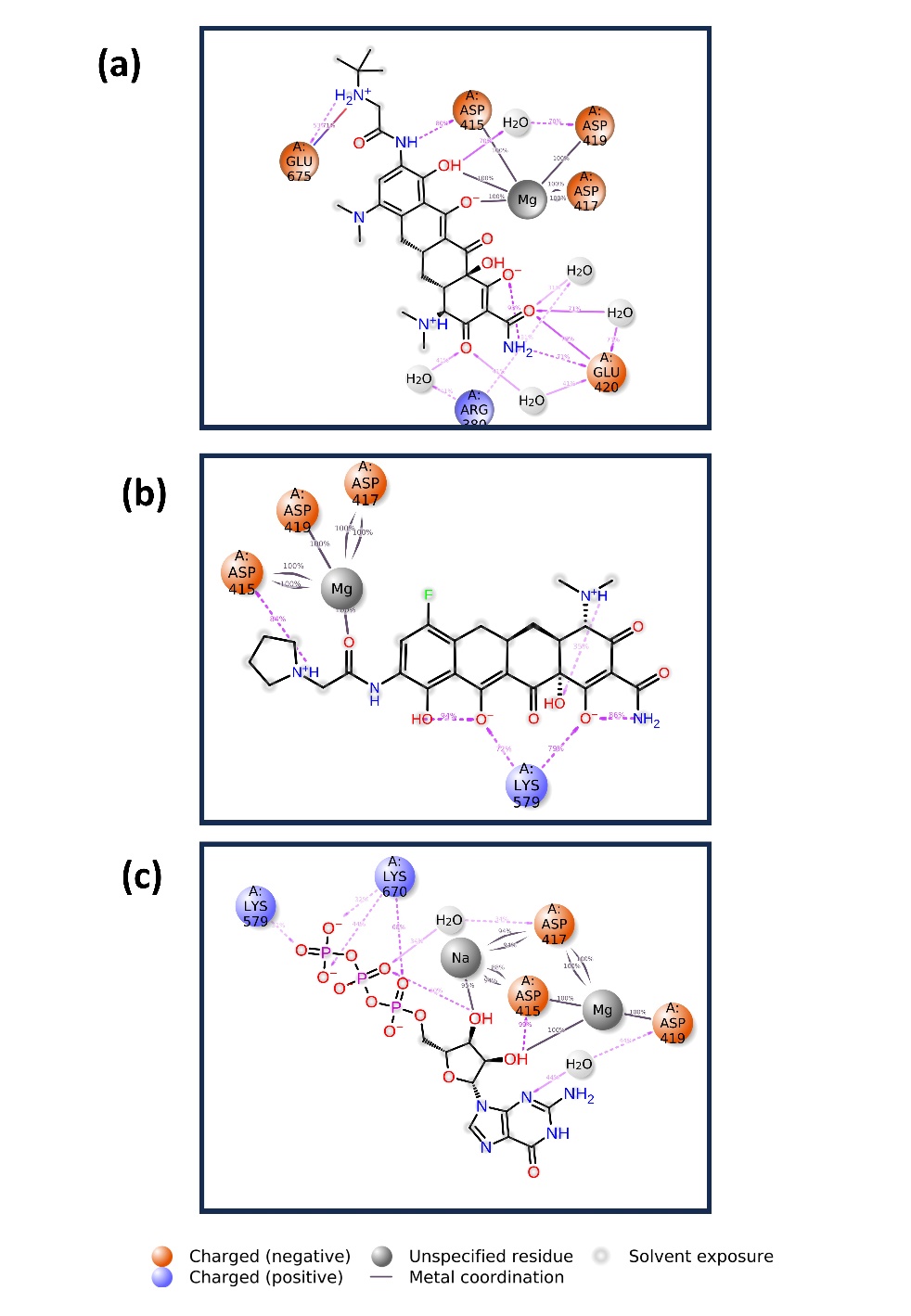


Supplementary Figure 5- Ligand contact schematic diagram of (a) Tigecycline, (b) Eravacycline and reference molecule (c) GTP extracted from the 100ns MD simulation trajectory of MPXV DdRp protein.


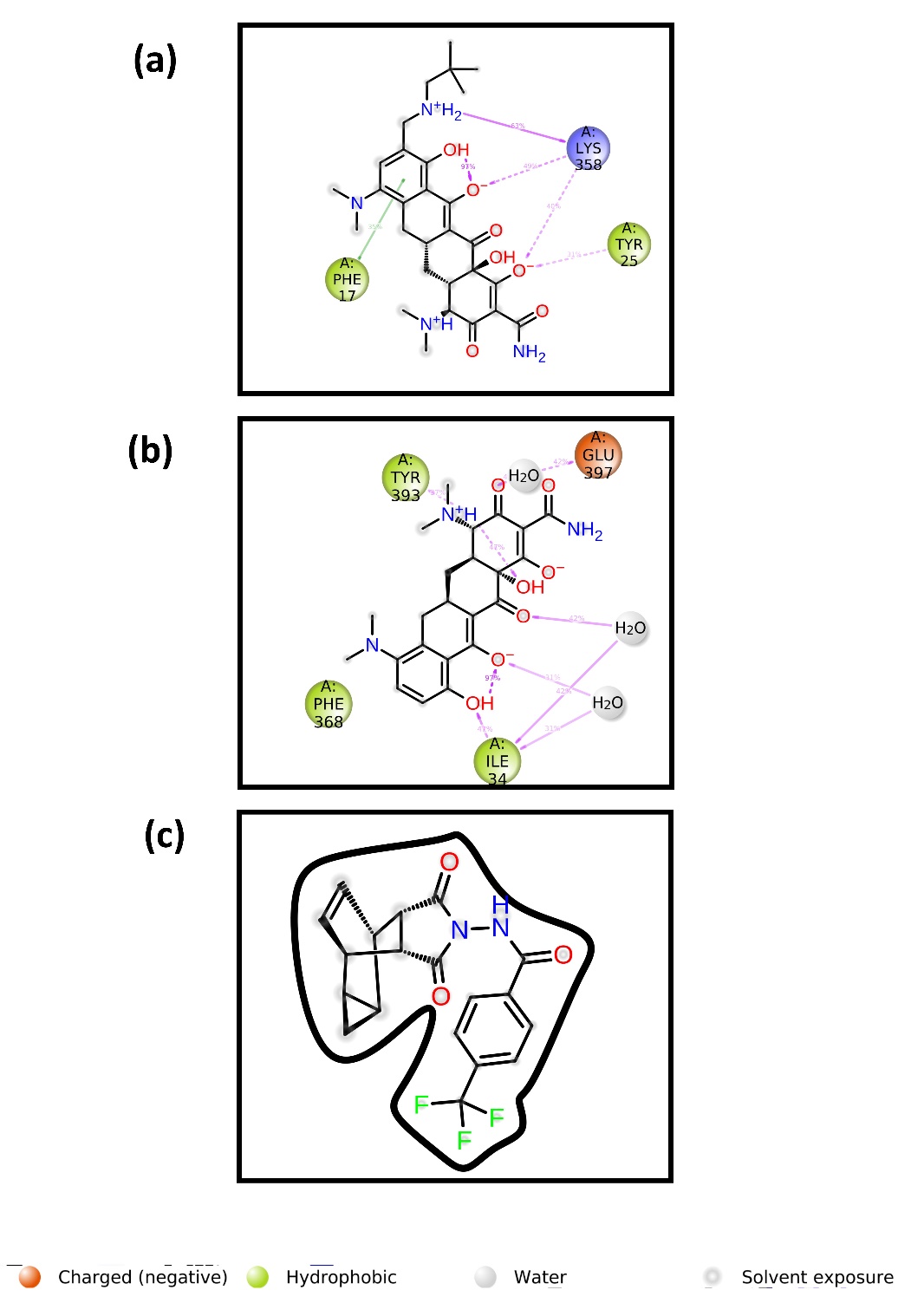
Supplementary Figure 6- Ligand contact schematic diagram of (a) Omadacycline, (b) Minocycline and reference molecule (c) Tecovirimat extracted from the 100ns MD simulation trajectory of MPXV cysteine proteinase protein.

3. Free binding energy calculation

3.1 Supplementary Table 3- Total Molecular Mechanics Generalized Born Surface Area (MM/GBSA) binding free energy (kcal/mol) values computed for the MPXV DdRp docked with the top two screened compound complexes and reference compound.

| Energy  components | Tigecycline | Evaracycline | GTP (Reference compound) |
| --- | --- | --- | --- |
| ∆G Bind | 36.42±10.69 | 47.96±5.59 | 8.02±18.63 |
| ∆G Bind Coulomb | -38.70±17.76 | 3.57±11.19 | -120.00±103.25 |
| ∆G Bind Covalent | 4.37±3.09 | 1.47±0.74 | 0.31±1.16 |
| ∆G Bind Hbond | -3.12±0.56 | -2.49±0.17 | -6.81±0.98 |
| ∆G Bind Lipo | -8.93±0.69 | -4.22±0.26 | -1.46±0.42 |
| ∆G Bind Solv GB | 132.75±12.58 | 74.01±12.12 | 159.64±83.70 |
| ∆G Bind vdW | -49.95±5.38 | -24.38±2.32 | -22.04±4.25 |
| Lig Strain Energy | 8.55±0.91 | 2.05±0.74 | 4.71±1.67 |

3.2 Supplementary Table 4- Total Molecular Mechanics Generalized Born Surface Area (MM/GBSA) binding free energy (kcal/mol) values computed for the MPXV proteinase docked with the top two screened compound complexes and reference compound.

| Energy  components | Omadacycline | Minocyline | Tecovirimat (Reference compound) |
| --- | --- | --- | --- |
| ∆G Bind | -30.68±2.23 | -42.47±4.77 | -18.18±3.31 |
| ∆G Bind Coulomb | 9.84±2.23 | -10.00±10.26 | -3.99±4.13 |
| ∆G Bind Covalent | 1.26±2.12 | 2.50±1.32 | 0.64±0.65 |
| ∆G Bind Hbond | -1.42±0.57 | -1.85±0.87 | 0.15±0.25 |
| ∆G Bind Lipo | -11.03±2.08 | -18.05±1.77 | -5.50±2.18 |
| ∆G Bind Solv GB | 10.96±5.46 | 22.47±7.63 | 12.17±3.82 |
| ∆G Bind vdW | -39.87±2.08 | -37.24±3.44 | -21.32±2.57 |
| Lig Strain Energy | 2.55±1.52 | 2.59±0.75 | 1.06±0.74 |
